# Directed information exchange between cortical layers in macaque V1 and V4 and its modulation by selective attention

**DOI:** 10.1073/pnas.2022097118

**Published:** 2021-03-15

**Authors:** Demetrio Ferro, Jochem van Kempen, Michael Boyd, Stefano Panzeri, Alexander Thiele

**Affiliations:** ^a^Neural Computation Laboratory, Istituto Italiano di Tecnologia, 38068 Rovereto, Italy;; ^b^Center for Mind and Brain Sciences, University of Trento, 38068 Rovereto, Italy;; ^c^Center for Brain and Cognition, Universitat Pompeu Fabra, 08002 Barcelona, Spain;; ^d^Department of Information and Communication Technologies, Universitat Pompeu Fabra, 08002 Barcelona, Spain;; ^e^Biosciences Institute, Newcastle University, NE1 7RU Newcastle upon Tyne, United Kingdom

**Keywords:** attention, feedforward processing, feedback processing, laminar interaction

## Abstract

Achieving behavioral goals requires integration of sensory and cognitive information across cortical laminae and cortical regions. How this computation is performed remains unknown. Using local field potential recordings and spectrally resolved conditional Granger causality (cGC) analysis, we mapped visual information flow, and its attentional modulation, between cortical layers within and between macaque brain areas V1 and V4. Stimulus-induced interlaminar information flow within V1 dominated upwardly, channeling information toward supragranular corticocortical output layers. Within V4, information flow dominated from granular to supragranular layers, but interactions between supragranular and infragranular layers dominated downwardly. Low-frequency across-area communication was stronger from V4 to V1, with little layer specificity. Gamma-band communication was stronger in the feedforward V1-to-V4 direction. Attention to the receptive field of V1 decreased communication between all V1 layers, except for granular-to-supragranular layer interactions. Communication within V4, and from V1 to V4, increased with attention across all frequencies. While communication from V4 to V1 was stronger in lower-frequency bands (4 to 25 Hz), attention modulated cGCs from V4 to V1 across all investigated frequencies. Our data show that top-down cognitive processes result in reduced communication within cortical areas, increased feedforward communication across all frequency bands, and increased gamma-band feedback communication.

## Significance

Attention is thought to modulate sensory processing by changing communication between cortical areas within specific frequency bands. Using local field potential recordings, we tested this influential model through laminar recordings in macaque V1 and V4. Attention modulated communication unexpectedly. In V1, it decreased communication across spectral frequencies except for granular-to-supragranular interactions. In V4, it increased communication across all spectral frequencies. Critically, attention increased V1–V4 feedforward communication across all frequency bands, decreased V4–V1 feedback communication in low-frequency bands, and increased beta and gamma feedback communication. These findings challenge existing theories of frequency specificity of feedforward and feedback interactions.

Goal-directed behavior requires the brain to integrate sensory information with cognitive variables. In neocortical areas, sensory information is conveyed by feedforward connections, while feedback connections convey information about cognitive states and goals. Feedforward and feedback connections rely on separate anatomical pathways and have been proposed to map onto distinct frequency bands of neural population activity (1–7). It is, however, unknown whether these signals differ across laminae, or how they are communicated between laminae within and between cortical areas.

Feedforward connections predominantly terminate in layer IV of sensory cortical areas. This information is passed on to layers II/III and further to layers V/VI, where recurrent inputs to layer II/III arise (8–11). Cognitive variables affect sensory processing through feedback connections, which predominantly terminate in layer I and V (12), but this termination pattern varies depending on hierarchical distances between areas (13). Feedforward and feedback signals have been proposed to show separate local field potential (LFP) spectral signatures. Feedforward signals have been associated with low-frequency theta- (1, 7) and gamma-band activity, originating and dominating in supragranular layers (1–7). Feedback signals have been associated with lower-frequency (alpha, beta) band activity, prominent in infragranular layers across the cortical hierarchy (1–4, 7, 14), although attention-related feedback signals in the gamma-frequency band between frontal eye field (FEF) and V4 have been reported (15). Alpha-related feedback has been linked to active inhibition (16, 17), suggesting that feedback signals, induced by attention to receptive field (RF) locations, should result in reduced alpha power. This occurs in infragranular layers in visual areas (18), but can also be less layer-specific (2). It is thus questionable whether feedback information is transmitted by alpha frequencies because attention, employing feedback, shunts alpha oscillations. In extrastriate sensory areas, attention increases LFP power in the gamma-frequency band (14, 15, 19–21), while, in primary visual cortex, attention can increase or decrease LFP power in the gamma-frequency band (1, 14, 21). Many of the above results were obtained by methods which do not provide insight into how these signals differ between laminae within an area, or between laminae across different areas. Thus, it remains unclear whether layer differences in these signals between cortical areas exist, and whether they are differently affected by cognitive goals.

To understand how information within and between areas is conveyed as a function of cognitive task, we performed simultaneous laminar recordings in areas V1 and V4 using 16-contact laminar probes while macaque monkeys performed a feature-based spatial attention task (22). We quantified communication between laminae and areas by measuring Granger causality (GC) using locally referenced LFP signals.

## Results

**Fig. 1.**
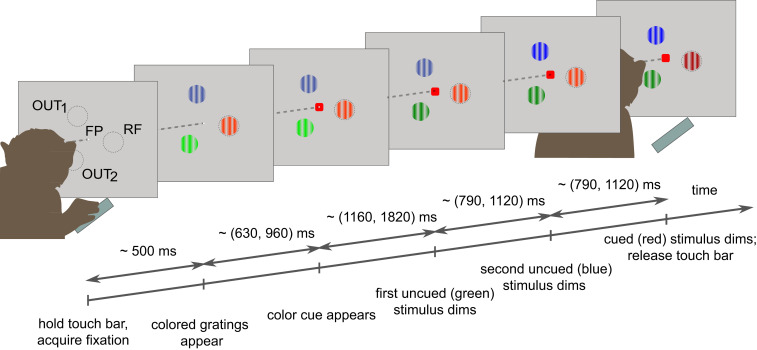
Behavioral task and recording setup. Covert, feature-guided visuospatial attention task. Monkeys fixated an FP and held a touch bar. Following fixation, three colored grating stimuli were presented equidistant to the FP: one stimulus covered RF locations and the other two were located outside the RF (OUT_1_ and OUT_2_). With a random delay from stimuli presentation, a colored attention-directing cue was presented at the FP indicating which stimulus was relevant on the current trial. Following the cue, the stimuli sequentially dimmed at unpredictable delays. When the relevant stimulus dimmed, the monkey had to release the touch bar to receive a fluid reward. Stimuli and cue colors, as well as the order of dimming of colored stimuli, were randomized across task trials. Ranges on the timeline indicate the range of random event delays. Circles outlining RF, OUT_1_, and OUT_2_ locations were not shown on screen.

Monkeys performed a covert, top-down, feature-guided spatial attention task. On each trial, attention was directed by a central colored cue to one of three possible locations in a pseudorandomized manner (Fig. 1). Monkeys had to detect a stimulus change at the cued location and ignore changes at uncued locations. To investigate how spatial attention affects interactions within cortical columns and between cortical columns, we simultaneously recorded LFPs from area V1 and V4 in two adult male monkeys (16-channel laminar probes, 150-µm intercontact spacing; 34 sessions for monkey 1, 28 for monkey 2). LFPs, rather than single-unit data, were used to assess information flow between populations because they capture local activity over a wide range of frequencies and are typically less variable than single-unit activity. We inserted probes perpendicular to the cortical surface (*SI Appendix*, Fig. S1*A*). The depth of recording contacts relative to cortical layers was determined by computing the LFP current source density (CSD; *SI Appendix*, Fig. S1*B*) (23) and the multiunit response latency (24). The earliest current sink of the CSD and the shortest multiunit response latency (multiunit activity envelope [MUA_E_]) identified input layer IV (*SI Appendix*, Fig. S1*B*). Recording sites superficial to the input-layer contacts were defined as supragranular layers (L I/II/III), and deeper sites were defined as infragranular layers (L V/VI; exact assignments described in *Methods*) (2, 3). For the vast majority of sessions, V1 and V4 RFs overlapped (detailed in the *SI Appendix* and *SI Appendix*, Fig. S2), although center-to-center RF positioning could be offset in some sessions.

LFPs were analyzed in different time windows. We mostly present data from LFPs in the time window preceding the first stimulus dimming (−503.25 to 0 ms, 512 time points). This corresponds to the period when attention was focused on the relevant stimulus, and when attentional modulation of spiking activity was most profound (*SI Appendix*, Fig. S11) (25, 26). We used bipolar rereferencing to improve spatial specificity of LFPs (*Methods* and *SI Appendix*).

**Fig. 2.**
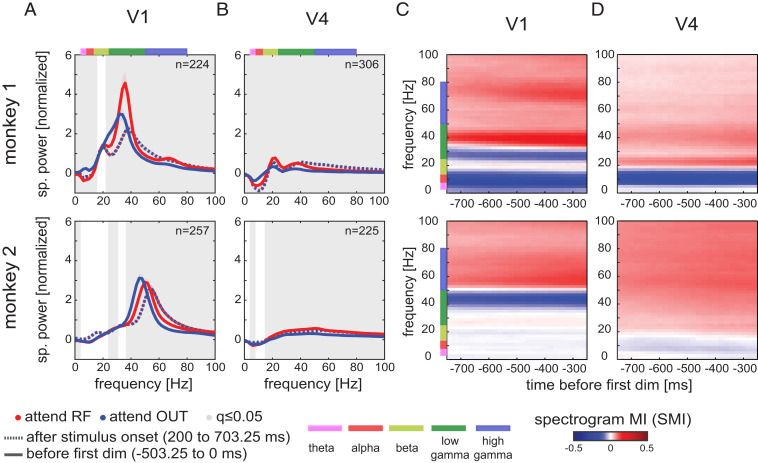
Attention decreases spectral power at lower frequencies and increases power at higher frequencies. (*A*) Spectral power (mean ± SEM across sessions and depths) of bipolar LFP signals in 503.25-ms task-related time windows. Dashed lines show spectral power after stimulus onset (200, 703.25 ms); solid lines show spectral power at times (−503.25, 0 ms) before first dimming; shaded areas show SEM. Frequencies with significant difference between attentional conditions at times before first dimming are shown by gray background (two-sided Wilcoxon signed-rank tests, FDR-corrected *q* ≤ 0.05). (*B*) Same as in *A*, but for V4 LFPs. (*C*) LFP attention SMI (mean across sessions and depths) for LFPs from monkey 1 (*Top*) and monkey 2 (*Bottom*). Spectral analysis was applied to 503.25-ms time windows sliding in 20-ms steps at times (−1,006.5, 0 ms) before the first dimming. (*D*) Same as in *C*, but for V4. Color bars at top and side of panels indicate the key frequency bands analyzed, with associated labels at the bottom.

LFPs were also decomposed into different frequencies using Fourier analysis (*Methods*). While these decompositions are well established and have shown robust frequency-specific differences in neural activity corresponding to different behavioral states or cognitive tasks, such frequency-specific power may capture both genuine and temporally extended narrow-band oscillations as well as broadband and nonstationary phenomena (27, 28). In line with previous reports (1, 14), location of spectral power peaks differed between animals (Fig. 2 and *SI Appendix*, Figs. S3 and S4). Despite this, key analyses were performed within frequency ranges widely used in the literature (1, 2, 14, 29), namely theta 4 to 8 Hz, alpha 8 to 13 Hz, beta 13 to 25 Hz, low gamma 25 to 50 Hz, and high gamma 50 to 80 Hz frequency. Since key spectral features varied across monkeys, these power peaks might fall into different bands relative to the above fixed frequency ranges. To account for this possibility, we also analyzed frequency bands aligned to the key features of individual monkey spectra. This approach yielded qualitatively similar outcomes for all results described, with key results reported in *SI Appendix*, *Supplementary Materials* and Fig. S11).

### Spectral Power and Coherence Across V1 and V4 Layers.

In V1, stimulus presentation increased spectral power relative to prestimulus power, across cortical layers at beta-band frequencies and above (*P* < 0.001 for beta and gamma bands for monkey 1, *n* = 224 pooled contacts; *P* < 0.001 for all frequency bands for monkey 2, *n* = 257; two-sided Wilcoxon signed-rank test; Fig. 2*A* shows data pooled across layers; *SI Appendix*, Figs. S3 and S5, show layer-resolved results). Attending to the RF increased low gamma-frequency peak power in monkey 1 across all layers when compared to attend-out conditions (Fig. 2*A* and *SI Appendix*, Fig. S3). An increase in low gamma-frequency peak power was not seen in monkey 2 (Fig. 2*A* and *SI Appendix*, Fig. S3). However, in both monkeys, attending to the RF stimulus resulted in 3 to 4 Hz higher low-gamma power peak location compared to attend-away conditions (changes were 32.82 ± 0.30 [SEM] Hz to 35.58 ± 0.26 Hz in monkey 1 and 46.83 ± 0.15 Hz to 50.63 ± 0.15 Hz in monkey 2; *P* < 0.001, both monkeys; *n* = 224 for monkey 1, *n* = 257 for monkey 2; two-sided Wilcoxon signed-rank test; Fig. 2*A*). This phenomenon has been described as a shift toward higher frequencies with attention (14), but it is better described as a drop in frequencies when attention is directed away from the RF, as stimulus presentation resulted in a gamma peak slightly higher than that induced by attention (Fig. 2*A*, dashed lines). Attention to the RF resulted in significantly higher spectral power at frequencies above the average of attend-RF and attend-out peak frequency location (*P* < 0.001 for monkey 1, *n* = 224; *P* < 0.001 for monkey 2, *n* = 257; two-sided Wilcoxon signed-rank tests) and significantly lower power below the average frequency (*P* < 0.001 in beta band for monkey 1, *n* = 224; *P* < 0.001 in low-gamma band for monkey 2, *n* = 257; two-sided Wilcoxon signed-rank tests; Fig. 2*A*). Additionally, decreases in V1 LFP spectral power with attention were found at lower frequencies (*P* < 0.001 for theta and alpha bands in monkey 1, *n* = 224; *P* < 0.05 in alpha band in monkey 2, *n* = 257; two-sided Wilcoxon signed-rank test; Fig. 2*A*). Spectral signatures of attentional modulation differed only slightly between cortical layers in V1 (*SI Appendix*, Figs. S3 and S5).

Stimulus onset reduced low-frequency spectral power in V4 in monkey 1 but increased it in monkey 2 (<13 Hz, *P* < 0.001 in theta and alpha bands relative to prestimulus power; two-sided Wilcoxon signed-rank tests; *SI Appendix*, Figs. S4 and S5). In both monkeys, stimulus onset increased spectral power for higher frequencies (>13 Hz, beta and gamma bands; *P* < 0.01 in monkey 1 beta band, *P* < 0.001 in monkey 1 low- and high-gamma bands, *n* = 306; *P* < 0.001 in beta and gamma bands for monkey 2, *n* = 225; two-sided Wilcoxon signed-rank tests). Attention to the RF stimulus resulted in significant increases in LFP spectral power in intermediate and high frequencies (from beta to gamma band; *P* < 0.001 in both monkeys, *n* = 306 contacts in monkey 1, *n* = 225 contacts in monkey 2; two-sided Wilcoxon signed-rank tests) and significant decreases at low frequencies (*P* < 0.001 in theta and alpha bands for monkey 1, *n* = 306; *P* < 0.001 in theta band for monkey 2, *n* = 225; two-sided Wilcoxon signed-rank tests; Fig. 2*B* and *SI Appendix*, Figs. S4 and S5). In V4, effects of attention on spectral power were similar across cortical layers in both monkeys (*SI Appendix*, Figs. S4 and S5).

To assess attentional modulation of spectral power relative to the time of cue onset and to the time of the first dimming, we calculated spectrogram modulation indices (SMIs) using a sliding window of 512 time points (503.25-ms length; *Methods*). Attentional modulation of spectral power (either positive or negative) increased after cue onset and persisted until the time of first dimming (*SI Appendix*, Figs. S3–S5). In V1, SMIs were positive for higher gamma frequencies and showed negative SMI for a narrow frequency range just below the average gamma peak, followed by positive SMIs in the beta band and negative SMIs in low-frequency ranges (alpha and theta bands; Fig. 2*C*). In V4, SMIs were negative for low-frequency spectral power, i.e., attention reduced low-frequency power in V4, while they were positive for frequencies >15 to 20 Hz, i.e., attention increased spectral power for medium and high frequencies (Fig. 2*D*). Attention-induced differences in spectral power were not a consequence of the presence of microsaccades on individual trials or of different rates or directions of microsaccades associated with different attention conditions (*SI Appendix*, Fig. S13 and *Supplementary Materials*).

**Fig. 3.**
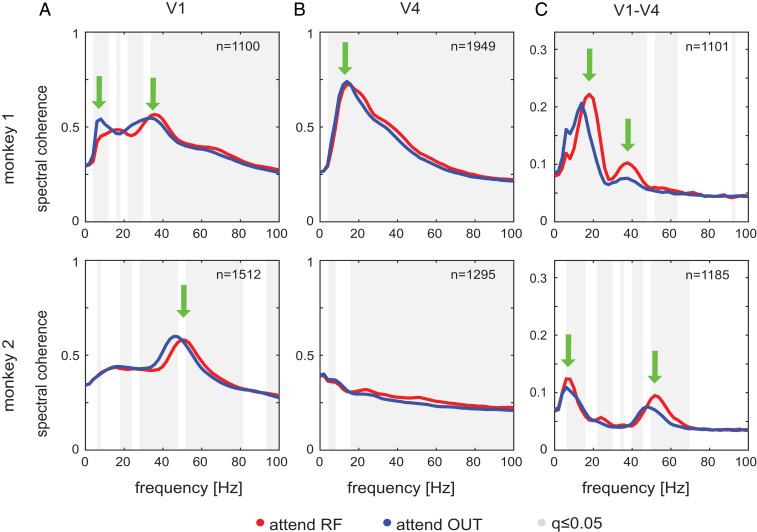
Effect of attention on LFP spectral coherence. (*A*) LFP spectral coherence across V1 depths (mean ± SEM across sessions and depth pairs) at times (−503.25, 0 ms) before the first dimming for monkey 1 (*Top*) and monkey 2 (*Bottom*). Gray background shows frequencies with significant difference between attentional conditions (two-sided Wilcoxon signed-rank tests, FDR-corrected *q* ≤ 0.05). (*B*) Same as in *A*, but for V4. (*C*) Same as in *A*, but for V1–V4 coherence. Green arrows indicate key spectral lobes mentioned in the text.

Attentional modulation of intraareal LFP (field–field) spectral coherence largely followed the pattern described for spectral power (Fig. 3 *A* and *B*). This indicates that the local (bipolar referenced) LFP power at specific frequencies is tightly coupled between layers. Attention to the RF resulted in significantly (∼1 to 2 Hz) higher spectral coherence peak locations in the gamma band in V1 (increase from 35.53 ± 0.13 [SEM] Hz to 36.50 ± 0.12 Hz in monkey 1, from 47.53 ± 0.06 Hz to 49.61 ± 0.06 Hz in monkey 2; *P* < 0.001 in both monkeys, *n* = 1,100 contact pairs for monkey 1, *n* = 1,512 for monkey 2; two-sided Wilcoxon signed-rank tests; Fig. 3*A*), increased spectral coherence at higher frequencies (*P* < 0.001 in low and high gamma in monkey 1; *P* < 0.001 in high gamma in monkey 2; two-sided Wilcoxon signed-rank tests; Fig. 3*A*), and decreased coherence at lower frequencies (*P* < 0.001 in theta and alpha bands, *P* < 0.05 in beta band for monkey 1; *P* < 0.001 in beta and low-gamma bands in monkey 2; two-sided Wilcoxon signed-rank tests; Fig. 3*A*). Slight increases were also found in lower bands (*P* < 0.05 in lower-beta band within ∼16 to 18 Hz in monkey 1; *P* < 0.001 in theta band for monkey 2; Fig. 3*A*). In V4, spectral coherence was increased by attention at higher frequencies (beta and gamma bands, *P* < 0.001 in both monkeys; *n* = 1,949 contact pairs in monkey 1, *n* = 1,295 in monkey 2; two-sided Wilcoxon signed-rank tests; Fig. 3*B*) and decreased at lower frequencies (*P* < 0.001 in theta and alpha bands in monkey 1; *P* < 0.01 in theta band in monkey 2; two-sided Wilcoxon signed-rank tests; Fig. 3*B*). Interareal spectral coherence showed three main peaks (Fig. 3*C*). One peak occurred at low frequencies (theta/alpha band), where attentional modulation differed between monkeys for the theta but not for the alpha band (coherence was decreased in theta band for monkey 1 [ *P* < 0.001, *n* = 1,940], increased in alpha band for monkey 1 [*P* < 0.001, *n* = 1,940], and increased in theta band [*P* < 0.05] and alpha band [*P* < 0.001] for monkey 2 [*n* = 1,802]; two-sided Wilcoxon signed-rank tests). A second peak occurred in the beta band, with increased coherence for attend-RF conditions (*P* < 0.001 in both monkeys; two-sided Wilcoxon signed-rank tests). A third peak occurred in the gamma band, which increased power for attend-RF conditions (*P* < 0.001 in low gamma for both monkeys, *P* < 0.001 in high gamma in monkey 1; *P* < 0.001 in monkey 2; two-sided Wilcoxon signed-rank tests; Fig. 3*C*). The effects of attention on spectral coherence were largely similar between layer pairs within areas, as well as across layer pairs between areas (*SI Appendix*, Fig. S6). In addition to causing shifts of intraareal coherence peaks (Fig. 3 *A* and *B*), attention also caused shifts in interareal coherence peaks. These were most evident for beta-band spectral peaks in monkey 1 and for gamma-band peaks in monkey 2 (Fig. 3*C*).

### Causal Communication Between Cortical Layers and Between Cortical Areas.

**Fig. 4.**
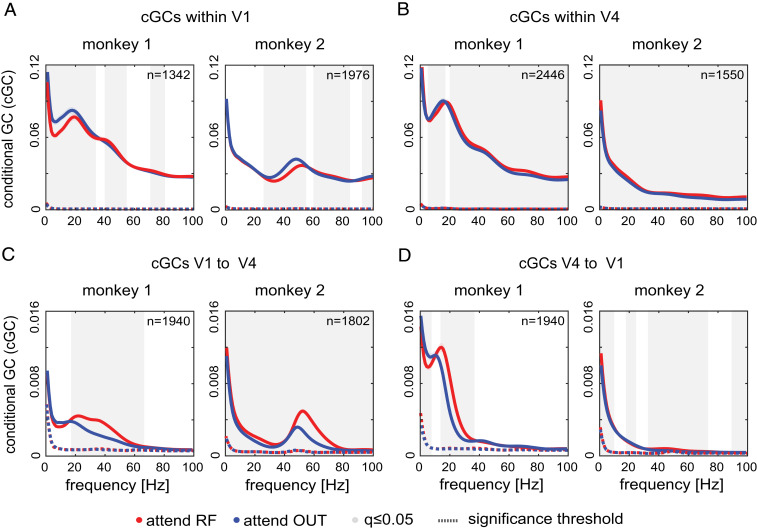
cGC for LFP signals. (*A*) Conditional cGC pooled across depths in V1 (mean ± SEM across sessions and directed depth pairs) at times (−503.25, 0 ms) before first dimming for monkey 1 (*Left*) and monkey 2 (*Right*). Gray background shows frequencies with significant difference between attentional conditions (Wilcoxon signed-rank tests, FDR-corrected *q* ≤ 0.05). (*B*) Same as in *A*, but for V4. (*C*) Same as in *A*, but for cGC from V1 to V4. (*D*) Same as in *A*, but for cGC from V4 to V1.

To determine the flow of information within and between layers within and between areas, we calculated conditional GC (cGC) (30). We first describe dominant interactions between layers and areas, irrespective of the effects of attention. This provides insight into which frequency bands predominantly carry feedforward and which frequency bands predominantly carry feedback information, independent of changing cognitive variables. Spectrally resolved intraareal and interareal cGCs averaged across contact pairs are shown in Fig. 4. All cGCs were significant (significance threshold is shown by dashed line in Fig. 4 *A*–*D*, computed as 95th percentile of cGCs with trials randomly shuffled; *Methods*).

**Fig. 5.**
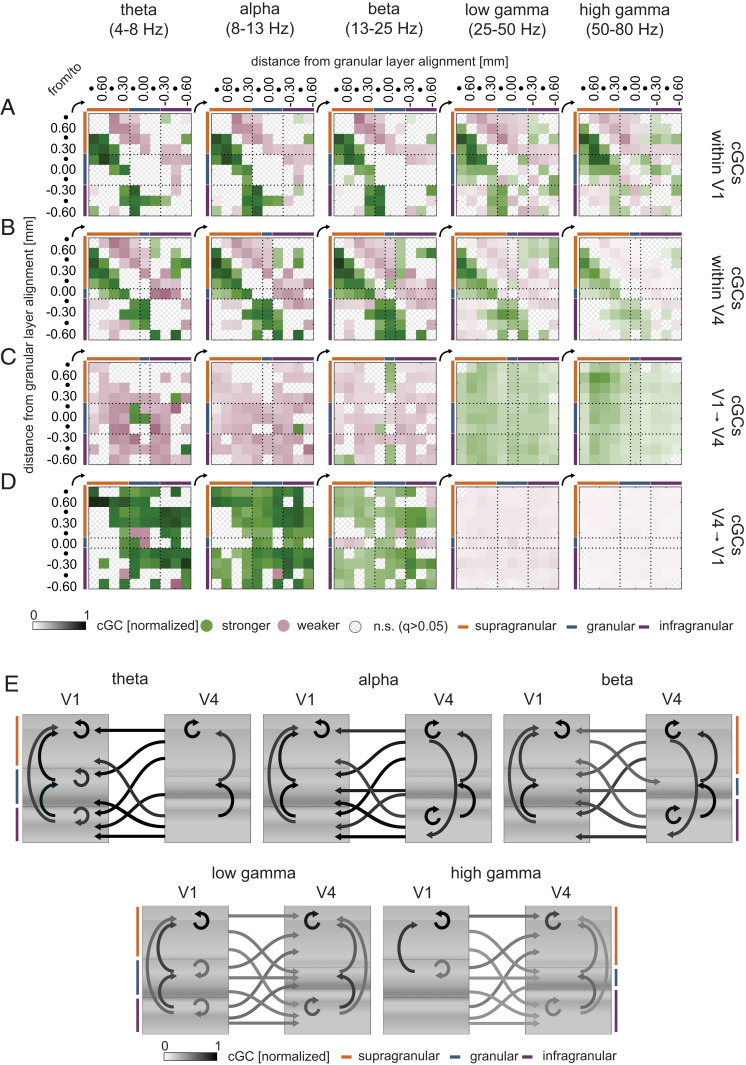
Directed connection matrices and influencer diagram of dominant cGC interactions before the first dimming. (*A*) Normalized cGC matrices (mean across sessions, pooled for the two monkeys) within V1 columns for different frequency bands. Connection matrices are color-coded to show significant dominant directions (green) and the opposite weaker directionality (magenta). Color intensity shows the relative cGC strength. Significance of cGC difference was assessed by two-sided Wilcoxon signed-rank tests, FDR-corrected (*q* ≤ 0.05) within frequency bands. cGCs were first normalized for each monkey to the peak magnitude across frequencies, then pooled. (*B*) Same as in *A*, but cGCs for V4. (*C*) Same as in *A*, but for V1-to-V4 pairs. (*D*) Same as in *A*, but for V4-to-V1 pairs. Numbers along the sides of panels indicate contact depth relative to the input layer (depth 0); dots show intermittent depths at 150-μm spacing. (*E*) Influencer diagram of significant dominant cGC interactions summarizing results in *A*–*D*. Arrows show dominant cGC interactions pooled for the two monkeys, averaged for the three laminar compartments (supragranular, granular, infragranular). Grayscale intensity of arrows indicates relative strength of cGCs (independently normalized for directions within V1, within V4, and between V1 and V4). Color bars show compartment assignment.

To plot cGC results, we normalized each cGC to the maximum cGC across the five frequency bands (separately for within-area and between-areas cGCs after averaging across all sessions) for each monkey. To assess the dominant directionality of communication, for each contact pair (X, Y), we determined whether cGC was stronger from X to Y, or whether it was stronger from Y to X, and whether the directional difference was significant for a given frequency range. We only present contact pairs where the directional cGC difference was significant (*q* ≤ 0.05, two-sided Wilcoxon signed-rank tests, false discovery rate [FDR]-corrected within frequency bands). In Fig. 5 *A*–*D*, significant differences are reported with color code indicating the dominant directions. For example, if cGC was larger in granular-to-supragranular direction than vice versa, it will be displayed in green, while the inverse direction will be displayed in magenta (Fig. 5 *A*–*D*; *SI Appendix*, Fig. S8, shows compartment-wise cGCs differences). Color intensity shows the relative strength of the interactions.

In V1, cGCs dominate in an upward direction within supragranular layers for all frequencies (Fig. 5*A* and *SI Appendix*, Fig. S8*A*), they dominate in an upward direction for all frequencies from granular to supragranular contacts, and they dominate in an upward direction from infragranular to granular and supragranular contacts in the theta-, alpha-, and beta-frequency range, with smaller directional differences in the gamma-frequency ranges. This pattern suggests that dominant interactions converge onto feedforward corticocortical output (supragranular) layers.

In V4 (Fig. 5*B* and *SI Appendix*, Fig. S8*B*), dominant interactions occurred in an upward direction within supragranular layers across all frequency bands. Additionally, dominant cGCs were present in an upward direction from granular to supragranular layers and from infragranular to granular layers. However, unlike in V1, cGCs dominated in a downward direction from supragranular to infragranular layers for most contacts and frequencies. Thus, within V4, a bidirectional dominance was found, whereby directly neighboring compartments communicated more strongly in an upward direction, while more distant compartments communicated more strongly in a downward direction.

Interactions between V1 and V4 dominated in the feedback direction in lower (theta to beta) frequency bands (Fig. 5 *C* and *D* and *SI Appendix*, Fig. S8 *C* and *D*) and in the feedforward directions in the gamma frequency ranges (Fig. 5 *C* and *D* and *SI Appendix*, Fig. S8 *C* and *D*). These V1–V4 interactions had little layer specificity with respect to origin or destination. While low-frequency theta feedforward V1-to-V4 interactions had the largest overall strength (of feedforward frequency bands; Fig. 4*C*), this directionality was still weaker than the feedback theta cGC strength.

cGC interactions from V4 to V1 were strongest in theta to beta frequency bands (Fig. 5*D* and *SI Appendix*, Fig. S8*D*). In the theta and alpha bands, they were most pronounced from V4 supragranular to all V1 layers. Strong interactions also occurred from V4 infragranular to V1 infragranular layer (Fig. 5*D* and *SI Appendix*, Fig. S8*D*). In comparison, V4-to-V1 cGCs in the gamma frequency ranges were small (even though they were significant). Thus, the feedback cGC interactions predominantly occurred in lower-frequency bands, and they originated in V4 supra- and infragranular layers and affected V1 supra- and infragranular layers.

These intra- and interareal cGC interactions are summarized in an “influencer” diagram (Fig. 5*E*). It shows that, in V1, dominant communication across almost all frequencies occurs in an upward direction toward the supragranular corticocortical output layer. In area V4, dominant communication occurs in a circular manner for lower frequencies (theta to beta), upward within compartments and between neighboring compartments but downward from supragranular layers onto infragranular layer. In the gamma frequency range, dominant V4 communications were directed upward toward the supragranular corticocortical output layer, mirroring the effects seen in V1. In the theta to beta frequency range, almost all interactions between V1 and V4 dominated in the feedback direction, while feedforward cGCs significantly dominated in the gamma frequency range.

### Attentional Modulations of cGC Interactions.

**Fig. 6.**
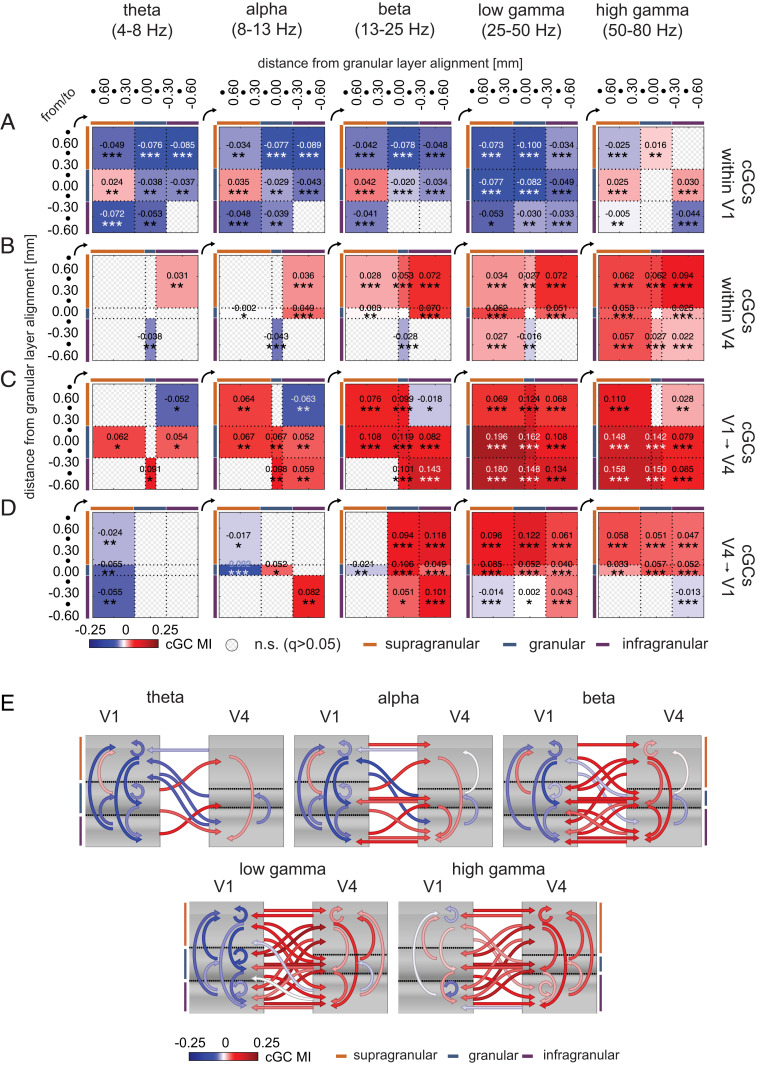
Attentional modulation of compartment-wise cGCs. (*A*) Significant attentional MI of cGC (cGC MI) among compartment pairs within V1 (mean across sessions, pooled for the two monkeys) at different frequency bands (significance assessed via two-sided Wilcoxon signed-rank tests, FDR-corrected within frequency bands; *q ≤ 0.05, **q ≤ 0.01, ***q ≤ 0.001). (*B*) Same as in *A*, but for V4. (*C*) Same as in *A*, but for cGC MIs from V1 to V4. (*D*) Same as in *A*, but for cGC MIs from V4 to V1. Numbers along the sides of panels indicate contact depth relative to the input layer (depth 0); dots show intermittent depths at 150-μm spacing. (*E*) Summary of main effects of attention on directed communication in different frequency bands. Arrows show significant attentional cGC MIs (mean across sessions, pooled for the two monkeys) for the three laminar compartments (supragranular, granular, infragranular; color bars along the sides). Color indicates whether attention increases directed communication (red) or decreases directed communication (blue) within and between areas.

To assess attentional modulation of cGCs, we calculated modulation indices (MIs) for each recording and determined whether MIs of cGCs between layer compartments were significant (*q* ≤ 0.05, two-sided Wilcoxon signed-rank tests, FDR-corrected within frequency bands; *Methods*). Fig. 6 shows significant intra- and interareal cGC attentional MIs pooled for the two monkeys averaged by laminar compartments (contact-wise attentional MI shown in *SI Appendix*, Figs. S9–S11). Surprisingly, within V1, cGC MIs were mostly negative, indicating that attention reduced cGCs in an upward and a downward direction across frequency bands (Fig. 6*A* and *SI Appendix*, Figs. S9*A*–S11*A*) except from granular to supragranular contacts. Additionally, high gamma-band cGCs increased with attention from granular to supragranular layers, from supragranular to granular contacts, and from granular to infragranular contacts (Fig. 6*A* and *SI Appendix*, Figs. S9*A*–S11*A*). The predominant reduction in cGC with attention within V1 was surprising given that attention increased neuronal firing rates across layers within V1 (*SI Appendix*, Fig. S12).

Attentional modulation of cGCs in V4 was very different than the pattern seen in V1 (Fig. 6*B* and *SI Appendix*, Figs. S9*B*–S11*B*). Across frequency ranges, it increased from supragranular to infragranular layers but decreased from infragranular to granular layers for theta to low gamma frequencies. This could enable feedback information to flow prominently to lower areas (supragranular V4 to infragranular V4 and onward to, e.g., V2, V1) while, at the same time, limiting potentially inhibitory interactions (assuming infragranular layers communicate inhibitory prediction signals) on stimulus-related processing (V4 infragranular to granular layers). In addition, downward communication (supragranular to infragranular) was increased by attention from theta to low gamma frequencies (Fig. 6*B* and *SI Appendix*, Figs. S9*B*–S11*B*). Attentional modulation of cGCs between compartments increased at higher frequencies, and gamma-band communication increased across almost all compartments.

Despite the reduction of cGCs by attention within V1, its influence on V4 increased across frequency bands for most compartment comparisons (Fig. 6*C* and *SI Appendix*, Figs. S9*C*–S11*C*). In lower-frequency bands, attention increased cGCs from V1 granular to almost all V4 layers (except for theta V1–V4 granular–granular interactions). However, in the theta to beta bands, V1 supragranular–to–V4 infragranular interactions were decreased. In the gamma-frequency bands, attention increased almost all V1-to-V4 interactions.

Attention decreased cGCs in the theta band from all V4 layers to V1 supragranular layers. In the alpha band, significant decreases occurred from granular and supragranular V4 to supragranular V1 (Fig. 6*D* and *SI Appendix*, Figs. S9*D*–S11*D*). In the beta and low gamma bands, attention increased V4-to-V1 cGCs in a downward direction (V4 supragranular to V1 granular and infragranular layers, from V4 granular to V1 granular and infragranular layers, and from V4 infragranular to V1 infragranular layers; Fig. 6*D* and *SI Appendix*, Figs. S9*D*–S11*D*). In the low-gamma range, it decreased the V4 infragranular–to–V1 supragranular interactions. In the high-gamma range, attention increased cGCs from V4 supragranular and from V4 granular layers to all V1 layers but decreased cGCs from V4 infragranular to V1 infragranular layers. Specific results for attentional modulation of cGCs obtained using frequency bands aligned to the key spectral features of individual monkeys are reported separately in *SI Appendix*, *Supplementary Materials* and Fig. S11. Critically, this splicing confirms the results presented here.

These patterns of attentional modulation are summarized in a frequency-dependent influencer diagram in Fig. 6*E*. It shows the attention-dependent reduction in cGCs across cortical layers and frequencies within V1, which nevertheless resulted in an increase in cGCs from area V1 to area V4. Feedback interactions were reduced by attention in the theta band, but mostly increased in the beta and gamma bands. Within V4, cGCs were mostly increased in the beta and gamma bands. Some of these interactions are predicted by established theories of frequency-specific interactions of feedforward and feedback connections, but many were in violation of established theory, as discussed in detail below.

## Discussion

Our investigations focused on LFP signal oscillations, their role in cortical processing, and how are they modulated by attention. We used cGC as a measure of causal communication among laminar LFP signals within V1 and V4 columns. LFPs measure extracellular signals, capturing local mass spiking and synaptic activity (31), allowing us to make predictions about the causal interactions of neural activity among laminae. The results challenge critical components of current models of cortical processing. Before we discuss this, we briefly summarize our main findings. Within V1, dominant communication streams are directed toward supragranular corticocortical feedforward outputs. Conversely, in V4, dominant communication was bidirectional, with one stream of supragranular corticocortical feedforward flow and a separate stream of supra- to infragranular feedback flow. Stimulus-driven feedforward communication from V1 to V4 dominated in theta and gamma frequency ranges, with little layer specificity. Stimulus-driven feedback communication from V4 to V1 dominated in the low-frequency range. Attention to the RF generally reduced communication between cortical layers in area V1, with a notable exception for granular-to-supragranular communication. Within area V4, attention predominantly increased communication in beta and gamma frequency ranges. Despite the attention-induced decrease of intraareal V1 communication, attention increased feedforward communication from V1 to V4 across frequency bands. Attentional effects on feedback communication (V4 to V1) differed between frequency ranges. Theta and alpha communication decreased while beta and gamma communication increased. Thus, feedforward interactions within and between cortical areas are neither limited to, nor dominant in, the gamma frequency range. Moreover, attention does not selectively increase gamma feedforward communication. Finally, feedback interactions between cortical areas, while dominant in the lower-frequency range, are generally decreased by attention at low frequencies, but increased by attention in the gamma band.

For V1 LFPs, spectral power peak locations in the gamma range differed between attend-RF and attend-away conditions. The peak location for attend-RF conditions resided at higher frequencies than for attend-away conditions (∼3 to 4 Hz). An equivalent result using electrocorticography surface recordings has been interpreted as a shift toward higher gamma peak frequencies induced by attention (14). However, our comparison with steady-state poststimulus gamma peak locations shows that attention keeps the peak gamma frequency closer to the stimulus-induced gamma frequency, i.e., attention stops it from dropping. This difference in interpretation is important, as it speaks to the role of attention and potential mechanisms involved. Attention affects normalization circuits, causing a concomitant increase in excitatory and inhibitory drive of the attended object/location (32–35). This increases the power and the frequency of gamma oscillations (34, 36). Attention thus ensures that stimulus representations remain sensory input-driven and sustained responses remain elevated (37–39). The predictive coding (PC) theory argues that predictable stimuli attain lower neuronal activation compared to less predictable stimuli. Within such a framework (PC; refs. 40, 41), the above could be interpreted in two ways. First, if attention reduced prediction generation for attended items, then prediction error coding populations would respond more strongly to sensory stimuli, as these are less predicted. This in turn increases feedforward communication, which has been associated with gamma frequency oscillations (2, 14, 42). Second, according to an extension of predictive coding that allows attentional signatures to arise naturally within the model (43, 44), attention increases the precision of predictions, making neurons respond more strongly to hidden causes (sensory input). Gamma power, as a signal of prediction errors (1, 40, 45), would thus be increased in superficial layers. Which of these two interpretations is correct remains to be determined.

We did not find consistent increases in gamma power with attention in V1 (only consistent differences in peak location were found). However, V4 gamma power and peak location were increased in both monkeys, in line with previous reports (14). Prominent gamma power and its modulation by attention have been argued to be largely confined to supragranular layers (5, 6, 18). We did not find major differences in absolute LFP gamma power, gamma power peak location, or attentional modulation of gamma power across supragranular, granular, or infragranular layers in either V1 or V4. Use of local bipolar referencing ensured that this was not an artifact of volume conduction. Hence, gamma-band activity is not restricted to superficial layers, and is thus unlikely to be a unique signature of feedforward interactions. This is in line with results reporting increased spike–spike coherence in the beta- and gamma-frequency bands in V4 infragranular layers (46).

Attention reduced LFP power in theta and alpha bands in areas V1 and V4, consistent with previous work (1, 2, 7, 18, 21), possibly linked to the reduction in low-frequency (<10 Hz) correlated variability that occurs in spiking activity in macaque V4 (47) and V1 (48). However, just as for gamma-band activity, these changes were not restricted to infragranular layers, but occurred across laminar compartments. These results equally question a strict separation between layer-specific frequency bands (5, 6, 49) and their potential association with feedforward and feedback signaling. They are more in line with recent reports about alpha sources across different modalities in primary sensory cortex (50).

### Communication Across Layers Within and Between Areas.

Interareal cGCs support the proposal that gamma- and theta-frequency interactions are strong in the V1-to-V4 feedforward direction (1, 2, 7, 14), while theta-, alpha-, and beta-frequency interactions are strongest in the feedback V4-to-V1 direction (1, 2, 7, 18, 21). However, cGCs within areas deviated from this scheme. While local feedback interactions from infragranular to granular layers and to supragranular layers were most prominent at low frequencies, strong and dominant low-frequency cGCs from granular to supragranular layers occurred. Moreover, dominant gamma cGC intraareal feedback communication was found (from infragranular to granular and to supragranular), contrary to the notion that this frequency band labels feedforward circuits (2). Thus, all cGCs in V1 dominate in a direction that targets the corticocortical output (supragranular) layers. This was the case for all frequencies, irrespective of the assumed role of oscillations in different frequency bands (1, 2, 29, 49, 51). It suggests that V1 is a distributor of feedforward information, with relatively less responsibility for feedback processing (as a consequence, it may have little effect in the generation of predictions; refs. 40, 43). The pattern changes slightly in V4, but it equally violates some key predictions about feedforward and feedback interactions. Namely, low-frequency cGCs dominated in the feedforward direction (supra- to infragranular layers), while gamma-band cGCs dominated in the feedback direction (infra- to supragranular layers).

Attention to the RF reduced almost all cGCs within area V1 except for low-frequency interactions from granular to supragranular layers. In the low gamma-frequency band, even those interactions were reduced. However, most interactions were increased in the high gamma-frequency band. The increase of cGCs from granular to supragranular layers is likely to boost feedforward output to other cortical areas, as expected from the increased efficacy of feedforward spiking interactions and thalamocortical interactions with attention (52, 53). If most intracolumnar feedback interactions served to compute context while spatial attention boosts elementary processing (at the expense of contextual processing), then these cGC reductions are expected. Low-frequency bands may predominantly play inhibitory roles (49, 51, 54). If these were reduced by attention, the increased firing rate seen in V1 in our and other studies (48, 53, 55, 56) would be a natural consequence. Within the predictive coding framework, it could be postulated that attention reduces the relative weight of predictions (although this is contrary to what was previously proposed; ref. 43). Intuitively, attending to stimuli from the external world could mean reshifting the balance from inferential to actuality processing, i.e., reducing the weight of internal priors. This would be achieved through reduction of feedback (local and interareal) and increase of feedforward processing. Such a reshifting has been shown to be mediated by acetylcholine (57, 58), which plays an important role in attention (25, 59, 60).

The attentional modulation of cGCs in V4 differed radically from that in V1. Attention increased theta- to beta-band cGCs from supra- to infragranular layers and reduced theta- to beta-band cGCs from infragranular to granular layers. In gamma bands, almost all cGCs were increased. V4 is a major recipient of feedback from attentional signals originating in FEF (15, 61). The feedback is excitatory and predominantly targets excitatory cells in layer II/III (62). It could explain why cGCs originating from V4 supragranular layers show the most pronounced increases with attention. However, it does not explain why it occurs across all frequencies if low-frequency interactions label inhibitory interactions. Our data suggest that an association with inhibitory roles is debatable for the case of FEF–V4 interactions, as we do not expect attention-mediated feedback to increase inhibition. The strong increases of cGCs between all layer compartments across frequency bands in V4 suggest that feedback and feedforward intracolumnar interactions within V4 do not strongly differentiate between frequencies.

Interactions from V1 to V4 were mostly increased by attention across frequency bands. Attentional increase was most profound in the gamma band, in line with the notion that gamma oscillations mediate feedforward communication (1, 2, 14). However, low-frequency interactions were also increased, which questions the generality of imputing feedforward communication exclusively to the gamma band.

Some of these directed causal influences measured in our study will be mediated indirectly through other areas, as direct reciprocal connections between V1 and V4 are comparatively sparse, especially in the feedback direction (63). Direct projections from V1 to V4 are largely restricted to the central 6° of the visual field (64, 65). This is where all our stimuli were presented, and some direct interactions would thus have been present. Key corticocortical interactions between V1 and V4 are mediated through V2 (66), but cortico-thalamic-cortical interactions could equally be important. The pulvinar regulates cortical synchrony in an attention-dependent manner, particularly in the low-frequency range (67), but it also affects gamma-frequency oscillations in V4 (68). The changes seen for V1-to-V4 cGCs in the low-frequency range could be mediated through cortico-pulvinar-cortical interactions (69, 70). On the one hand, this might explain the relative absence of layer specificity in cGC interactions between V1 and V4, irrespective of their direction. On the other hand, it has been argued that the cortico-pulvinar-cortical connections replicate the corticocortical pattern, whereby, e.g., feedback connections originate in deeper cortical layers, which, via pulvinar, modulate superficial layers in lower cortical areas (70, 71). Regardless of potential indirect mediations of reciprocal V1–V4 causal interactions, our data add important information about layer and frequency specificity of attention- and stimulus-related interactions, which, to the best of our knowledge, has not previously been investigated.

Attention reduced communication from V4 to V1 in the theta band and most strongly increased cGC in the beta band. Pronounced increases also occurred in the gamma band, demonstrating that feedback interactions also operate strongly in the gamma band. V4-to-V1 cGCs equally did not show strong laminar specificity. While this could be a consequence of subcortical routing (69, 70), it could also be a consequence of a termination pattern of V4 feedback that predominantly targets layer 1 dendritic spines through excitatory synapses (72, 73). These terminations can thereby influence pyramidal cells across supra- and infragranular layers. The predominance of excitatory connections on pyramidal cell dendrites is not consistent with the proposal that predictions generated at higher cortical levels act through disynaptic inhibition for messages passing to lower areas (40).

A recent theory of “predictive routing” (45) proposed that low-frequency feedback prepares feedforward pathways by inhibiting gamma and spiking activity associated with predicted inputs. A reduction in feedback (prediction) signals would thus cause disinhibition. Our results align, but also argue for an extension of this predictive routing scheme. We argue for different hierarchies of prediction generation: some are automatic (e.g., surround suppression, basic contour integration, contrast normalization), while others are associated with higher cognitive functions (e.g., working memory, feature search, spatial attention, value estimation). We also propose that these may employ different feedback networks. Automatic prediction generation mostly works within connections that affect nonclassical RF interactions. This would explain why cooling of higher-level areas results in reduced surround suppression (74), i.e., upon cooling, higher areas cannot pass predictions to lower areas. Inhibition is thus reduced, and prediction error (or, to use different words, sensory coding) signaling will be large. On the contrary, interactions between neurons sharing classical RF (cRF) locations counterbalance the prediction coding, i.e., they are predominantly excitatory. This explains why cooling of higher cortical areas results in reduced cRF responses (74). It is these cRF routes that might be exploited by higher cognitive functions, which, through a separate form of feedback, generate biased competition and simultaneously serve to suppress automatic predictive coding. Our data of attention-induced increased feedforward, but decreased feedback, communication within V1, increased feedforward and feedback cGCs within V4, and increased bidirectional communication between V1 and V4 (with overlapping cRFs) across most frequency ranges support such a proposal.

## Methods

### Experimental Procedures.

#### Animals and procedures.

We simultaneously recorded from visual areas V1 and V4 of two adult male rhesus macaque monkeys (*Macaca mulatta*, 10 to 11 y of age) while they performed a sustained top-down, feature-guided visuospatial attention task. Experimental procedures were in line with Directive 2010/63/EU of the European Parliament and the Council of the European Union, the Guidelines for Care and Use of Animals for Experimental Procedures from the National Institutes of Health, the Policies on the Use of Animals and Humans in Neuroscience Research from the Society for Neuroscience, the UK Animals Scientific Procedures Act, and the university animal care, welfare, and ethical review body. Animals were group-housed together in groups of two or three animals in same- or mixed-sex groups. Housing and husbandry complied with the guidelines of the European Directive (2010/63/EU) for the care and use of laboratory animals and followed the Animal Research Reporting of In Vivo Experiments principles on reporting animal research. Animals were motivated to engage in behavioral tasks through fluid control at levels that do not affect animal physiology and have minimal impact on psychological wellbeing (75).

#### Attention behavioral task.

Monkeys had to touch a lever for the appearance of a centrally placed fixation spot. Thereafter, they had to direct their gaze at a fixation point (FP) positioned at the center of the cathode-ray tube screen, with a fixation window of ±1 to 1.5 degrees of visual angle (DVA) throughout the trial duration.

At 500 ms after fixation onset, three colored, moving grating stimuli (stationary gratings for 19 of 28 recordings for monkey 2) occurred, located equidistant from the FP. One stimulus was centered on the RF of recorded cells in V1 and the other two were positioned outside (at locations OUT_1_ and OUT_2_). The RFs of recorded cells were mapped at the beginning of each experimental session (as detailed later and in the *SI Appendix*).

At 630 to 960 ms after stimulus onset (random delay, uniformly distributed, 1-ms steps), a colored cue was presented at the FP. The color of the cue instructed the monkey to monitor the stimulus of matching color (e.g., a red cue instructed the animal to monitor the red visual stimulus) for a change in luminance contrast and ignore changes at the other stimulus locations.

After a random delay, the three stimuli started to sequentially dim in a pseudorandom order. Delays for subsequent dimmings ranged between 1,160 and 1,820 ms (the first dimming could occur 1,160 to 1,820 ms after cue onset, the second dimming could occur 790 to 1,120 ms after the first dimming, etc.).

During the entire trial period, monkeys had to keep fixating the FP. Upon cued stimulus dimming, monkeys had to release the touch bar within 600 ms to receive a fluid reward. Fig. 1*A* graphically shows the time course of a sample trial of the main behavioral task. The grating stimuli had a diameter between 2 and 4 DVA, adjusted in accordance with the size and eccentricity of the recorded RFs. Stimulus spatial frequency was 1.5 cycles per DVA, with a temporal frequency of 1 cycle per second (in sessions where they moved) and an orientation of 30°. The stimulus color at a given location was fixed (red, green, or blue) for trials of the same session but randomized across sessions to cover all of the six possible color configurations. In the same way, the cue color (red, green, or blue), the order of dimming of the three stimuli (six possible dimming orders), as well as the direction of movement of the grating stimuli (two possible opposite directions where applicable) were pseudorandomized across trials to cover all possible task configurations. The dimmed stimulus was of a different luminance (*SI Appendix*, Table S1, shows color specifications) but very similar hue relative to the predimmed stimulus (undimmed International Commission on Illumination [CIE] *x*–*y* color coordinates, red [0.54, 0.42], green [0.24, 0.65], blue [0.15, 0.08]; dimmed CIE *x*–*y* color coordinates, red [0.54, 0.42], green [0.24, 0.64], blue [0.14, 0.08]). Thus, animals most likely have responded to a change in luminance, not hue.

There were 36 conditions in total, which comprised a so-called cycle. In each cycle, all 36 conditions would occur at least once, selected on a random basis. If the monkey performed the trial correctly, the condition was removed from the cycle pool. If the trial was not completed correctly, the condition was reinserted into the cycle pool and would be reselected on a random basis until all conditions had had been performed correctly.

#### Electrophysiological recordings.

The data were collected over 62 sessions (34 for monkey 1, 28 for monkey 2), yielding a total of 35,744 correct trials (15,892 in monkey 1, 19,852 in monkey 2). These were out of 36,912 total trials (16,698 in monkey 1, 20,214 in monkey 2) where monkeys kept fixation, yielding behavioral performances of 95.17% correct for monkey 1 and 98.21% correct for monkey 2 (disregarding fixation breaks).

#### Electrophysiological data analysis.

Signals were extracted in time windows relative to task-related events: after stimulus onset (0 to 503.25 ms, 512 data points; see below for LFP sampling frequency), after cue onset (0 to 503.25 ms, 512 data points), and before dimming times (503.25 ms before each of the three subsequent dimmings, 512 data points). Baseline activity time window started 200 ms before stimulus onset and covered up to 30 ms after stimuli onset.

Data were replayed offline, sampled with 16-bit, band-pass–filtered at 0.5 to 300 Hz, and down-sampled by a factor of 32 to a sampling frequency *Fs* = 1,107.375 Hz to obtain LFP data. Spiking activity was accessed by band-pass filtering between 600 and 9,000 Hz, then further analyzed both at the level of multiunit activity by extracting the MUA_E_ and by sorting single- and multiunit spiking waveforms (additional details provided in *SI Appendix*).

#### CSD analysis.

The CSD signal was obtained by applying the spline inverse CSD (iCSD) method (76). Starting from the direct equation for the field potential Φ generated by a point source *C* positioned at the origin of an isotropic medium Φ = *F*⋅*C*, the iCSD was estimated by inversion of the conduction matrix *F* as *Ĉ* = *F*^−1^⋅Φ. The coefficients of *F* were computed by electrostatic field equations for point sources by assuming that they are evenly distributed within isotropic cylindrical discs of finite radius *R* and by assuming smooth CSD variation along depth dimension. CSD variation along depths was approximated by cubic splines interpolation. In our computations, we assumed a disk radius *R* = 500 µm, and we used conductance σ = 0.4 S/m. The conductance term could affect the magnitude of iCSDs but not their spatial profile. The iCSD was filtered by a Gaussian filter with SD 200 μm along the depth dimension.

#### Laminar alignment.

Laminar signals from different experimental sessions were aligned to layer IV of both V1 and V4. Layer IV was identified for each session as the earliest current sink across laminae using CSD of LFPs and by analyzing the shortest latency of the stimulus-evoked MUA_E_ response (*SI Appendix*, *Supplementary Materials*). Based on their distance from reference coordinate, signals from the corresponding recording channels were assigned to three main laminar compartments: supragranular, granular, and infragranular. For V1, channels above the reference channel at distances of 0.25 to 1 mm were labeled as supragranular, channels above or below the reference channel within 0.25 mm were labeled as granular, and channels below reference at distance range 0.25 to 0.75 mm were labeled as infragranular. For V4, channels above the reference channel in the range 0.1 to 1 mm were labeled as supragranular, channels within 0.1 mm above or below the reference channel were labeled as granular, and channels below the reference channel at 0.1 to 0.75 mm were labeled as infragranular.

#### Spectral power.

The estimation of LFP signal power across frequencies was performed using a multitapering approach using the Chronux toolbox (77). We set the tapering to *K* = 3 Slepian waveforms with time–bandwidth product *TW* = 2 (*N* = 512, *T* = *N*/*F*_s_ = 503.25 ms, *W* ≈ 4 Hz). The LFP spectral power *S*_i_(λ) was normalized for each λ ∈ [0, *F*_s_/2] to baseline spectral power (minus trial-averaged baseline power, divided by the SD of baseline power). Baseline spectral power was obtained for the period of −512 to 0 data points before stimulus onset.

#### Spectral coherence.

The relationship between the spectral components of LFP signals recorded from multiple channels was quantified in terms of spectral coherence. This measure is computed by means of the cross-spectrum power density *S*_ij_(λ) = *X*_i_(λ)⋅*X*_j_*(λ) involving the spectral representations *X*_i_(λ) and *X*_j_(λ) and of signals in channels i and j. The spectral coherence is defined as *C*_ij_(λ) = |*S*_ij_(λ)|^2^/|*S*_i_(λ)⋅*S*_j_(λ)|, λ ∈ [0, *F*_s_/2]. The values assumed by *C*_ij_(λ) are in the range [0,1], where 0 means that the frequency components of the two signals are completely unrelated and 1 means the two signals have perfectly linear relationships at a given frequency component. The terms *S*_i_(λ), *S*_j_(λ), and *S*_ij_(λ) were computed with the use of the Chronux toolbox via multitaper estimation (using *K* = 3 Slepian sequences, *TW* = 2).

#### Time/frequency spectral modulation.

The spectral characterization was also performed in the time/frequency domain. LFP spectral power and coherence were both computed by using sliding time windows of duration 503.25 ms (*N* = 512 time points), shifted in time every 20 ms to cover 1,006.5 ms before the time of first stimulus dimming. The spectral resolution was Δ*f* = *F*_s_/*N* ≈ 2 Hz, and temporal resolution was Δ*t* = 20 ms.

#### GC analysis.

We measured directed causal communication between LFPs recorded at different contacts by using GC. GC is a multivariate directed measure that allows one to quantify the degree of causal relationship (or communication) between two nodes. For any directed contact pair (X,Y), we analyzed GC in a 503.25-ms time window (*N* = 512 time points at 1,017.375-Hz sampling rate) preceding the first dimming time. Details regarding GC calculation are provided in *SI Appendix*, *Supplementary Materials*.

#### Attentional MI.

To investigate the effects of attention, we compared results for the trials where attention was directed toward RF visual location against the ones where it was directed at outside locations OUT_1_ and OUT_2_. Since the LFP spectral characterization for these two latter cases did not show prominent differences, we combined them in a single attend-OUT condition by random-subsampling an equal number of trials with condition OUT_1_ and OUT_2_. The MI for the measure *F* (spectral power or cGC) was defined as *F*_MI_ = (*F*_RF_ − *F*_OUT_)/(*F*_RF_ + *F*_OUT_).

#### Statistical tests and significance.

Significance of the difference in spectral power, coherence, or cGCs (e.g., between time windows [before stimuli onset vs. after stimuli onset], attentional conditions [attend RF vs. attend OUT], or directionality of cGCs [f_X→Y|Z_ vs f_Y→X|Z_]), as well as the significance of attentional MIs (*F*_MI_s), were tested across experimental sessions by two-sided Wilcoxon signed-rank tests. Comparisons across all three attention conditions were conducted using a single-factor (condition) repeated-measures ANOVA. *P* values were corrected for FDR at *q* = 0.05 (78).

## Supplementary Material

Supplementary File

## Data Availability

Original LFP data and analysis code have been deposited in g-node and are available at https://gin.g-node.org/demetrio.ferro/V1-V4-LFPs-and-Visual-Attention and https://doi.gin.g-node.org/10.12751/g-node.824cgx/.
