# Supplementary material for: Directed information exchange between cortical layers in macaque V1 and V4 and its modulation by selective attention
Source: Proc Natl Acad Sci U S A. 2021 Mar 15;118(12):e2022097118. doi: 10.1073/pnas.2022097118 (PMC8000025; doi:10.1073/pnas.2022097118)
Supplement: Supplementary File [file pnas.2022097118.sapp.pdf]

PNAS
www.pnas.org

Supplementary Information for

Directed information exchange between cortical layers in macaque V1 and V4 and its modulation by selective attention

Demetrio Ferro[1,2,4], Jochem van Kempen[3], Michael Boyd[3], Stefano Panzeri[1,5] and Alexander Thiele[3,5+]

[1] Neural Computation Laboratory, Istituto Italiano di Tecnologia, Rovereto, Italy
[2] Center for Mind and Brain Sciences (CIMeC), University of Trento, Rovereto, Italy
[3] Biosciences Institute, Newcastle University, NE1 7RU, Newcastle upon Tyne, United Kingdom
[4]Center for Brain and Cognition, and Department of Information and Communication Technologies, Universitat Pompeu Fabra, Barcelona, Spain
[5] Senior and corresponding authors: stefano.panzeri@iit.it and alex.thiele@ncl.ac.uk
[+]Lead Contact

This PDF file includes:

Supplementary text
Figures S1 to S13
Tables S1 to S4
SI References

Supplementary Information Text

Supplementary Methods

Surgical preparation

Animals were implanted with a head post and recording chambers over area V1 and V4 under sterile conditions and general anaesthesia. Surgical procedures and postoperative care conditions have been described in detail previously (*1*).

Behavioral and data acquisition setup

Monkeys were trained to comfortably sit in a primate chair while being head stabilized by the cranial head holder. Stimuli were presented on a cathode ray monitor (22'' CRT, 120Hz, 1280x1024 pixel resolution) placed at 54 cm distance to the monkey's eyes. Eye position was calibrated and monitored by an eye tracking system operating at a sampling rate of 220Hz. Stimulus presentation and behavioral control was handled by Remote Cortex 5.95 (Laboratory of Neuropsychology, National Institute for Mental Health, Bethesda, MD).

Electrophysiological recordings were performed using passive laminar probes with 16 recording contacts, inter-contact spacing of 150 μm (ATLAS Neuroengineering, Belgium). The laminar probes were inserted perpendicularly to the cortical surface with the support of a hydraulic micromanipulator (NARISHIGE MO-97A, Japan). All contacts were initially referenced to a wire positioned either in the V1 chamber, or in the V4 chamber.

Data from the two chambers were simultaneously recorded using a digital acquisition and control system (Digital Lynx, Neuralynx, USA) with a sampling frequency of 32556 Hz (~32 kHz), at 24 bits.

Basic Response Characterization (Receptive Field Mapping)

Prior to starting the attention paradigm, the location and size of the RF was measured by a reverse correlation method (*2*).

From this, RF maps were initially estimated online to determine the stimulus locations in the attention paradigm. Offline RF analysis was done based on local population activity (envelope multi-unit activity, MUA_E (*3*)), using a time window from 50-150 ms after RF mapping stimulus onset. We also attempted to derive an LFP based RF estimate, however, these estimates were much less reliable than MUA_E estimates, with often very low signal to noise ratio, particularly for V4 recordings. Hence, RF estimates relied on MUAe signals. Receptive field mapping was performed with 0.5˚, 1˚ or occasionally 2˚ (V4 only) sized stimuli. This approach overestimates V1 RF sizes but provides a sufficiently accurate estimate of the RF location (which was the main aim for this study). It was sufficient to determine spatial location of V1 and V4 retinotopic representation (and overlap of RFs),

while minimizing the number of trials an animal performs before moving to the main task (and thus maximized the number of trials available in the main task, Fig. S2).

We did not test for feature selectivity in our recording sessions, as it is exceedingly difficult to match feature selectivity between V1 and V4 recording locations. Thus, separating according to matched vs. non-matched feature selectivity would result in diminishingly small sample sizes (along with the question, which feature to match, e.g. orientation, color, direction selectivity, etc). Critically, detailed characterization of feature selectivity prior to the main task reduces the number of trials available for analysis from the main task, due to satiation.

Multi-Unit Activity Envelope and Signal to Noise Ratio

MUA_E was computed as described in detail previously (*3*). The higher frequency signal component (600-9000 Hz) was down sampled by a factor of 4, full-wave rectified, low-pass filtered at 500 Hz (Butterworth, zero-phase digital filter of order 5), and further down sampled by a factor of 8 to a frequency of 1017.375 Hz.

Signal to Noise Ratio (SNR) computation was performed on MUA_E signals in $n = 8$ sliding time windows of length 50 ms shifted every 10 ms from 30 to 150 ms after stimuli onset. The SNR is computed as the maximum average magnitude of baseline corrected MUA_E signal across time windows, i.e. $\mathrm{SNR} = \max_n[(\langle s_n(t)\rangle - \langle b(t)\rangle)/\sigma_b]$, where $\langle s_n(t)\rangle$ is MUA_E average within time window n, and $\langle b(t)\rangle$ and σ_b are respectively the baseline mean and standard deviation.

Response Latency analysis

Response latency was calculated according to (*4*), assuming that stimulus responses arises at random times with a Gaussian distribution, and that a fraction of the response function dissipates exponentially after reaching a peak magnitude. On our data, the response function was estimated by fitting the 150 ms baseline-corrected post-stimulus MUA_E signals to a distribution $f(t)$ consisting in the sum of ex-Gaussian and cumulative Gaussian functions:

$$f(t) = d \cdot e^{\mu\alpha + (\sigma^2\alpha^2/2) - \alpha t} \cdot G(t, \mu + \sigma^2\alpha, \sigma) + \ c \cdot G(t, \mu, \sigma).$$

The parameters μ and σ respectively match the mean and standard deviation of the response function onset time when considering the response as non-dissipating. The parameter α is the dissipation rate, and the parameters c and d act as weighting factors for the response magnitude and dissipation terms. The functions $G(t, \mu', \sigma')$ are cumulative density functions of a generic normally distributed variable with mean μ' and standard deviation σ'.

Response latency is computed as the smallest time delay allowing to achieve 33% of the peak in the response magnitude $\hat{f}(t)$ estimated by least-square error minimization. In symbols, we computed latency as: $\mathrm{lat}_{33} = \min\left\{t \in T : \hat{f}(t) = 0.33 \cdot \max_{u \in T} \hat{f}(u)\right\}$. To reduce computational cost of the

least-square fit procedure, the empirical MUA_E response was smoothed by a moving average filter with length 5 samples, covering ≈5 ms.

Trials and Channels inclusion criteria

Our analyses included only trials with behaviorally successful outcome. To correct for eventual artifacts, which could be due to transient drifts of the probe, possibly caused by slight movements of the animal, we set a signal thresholding rule for trials selection. Trials were discarded if the baseline normalized signal energy was higher than the energy of a signal with magnitude 20 times bigger than baseline, i.e. if the signal $\bar{x}(t) = (x(t) - \langle b(t) \rangle)/\sigma_b$ had energy $\xi_{\bar{x}} > 20^2$, where $\xi_{\bar{x}} = \frac{1}{T}\int_0^T |\bar{x}(t)|^2 dt$, $x(t)$ is the LFP/MUA_E signal in any of the task-relevant time windows, $\langle b(t) \rangle$ and σ_b are the signal mean and standard deviation at baseline.

Applying this thresholding rule led to the rejection of 2.1% of the trials, hence to the selection of 34992 out of 35744 behaviorally correct trials (15468 were from monkey 1, 19524 for monkey 2).

In all analyses we ensured to use equal amount of trials per attentional condition (RF, OUT_1, OUT_2) by random sub-selecting trials in each session so that the amount of trials per condition was equal to the minimum amount of trials available in the three conditions.

To prevent signal contamination due to common grounding or strong remote signal sources, the signals for each electrode contact from the two cortical areas were locally referenced via bipolar differentiation. The signal from depth z_i was replaced by the difference between signals at depths z_{i+1} and z_{i-1}, as if it was recorded by a virtual electrode located at intermediate depth between its two neighboring contacts. This procedure did not allow to consider the two outermost channels (as they could not be re-referenced with respect to their neighbor channels), but this was often not problematic as the channels located at outer positions were usually outside the grey matter of the targeted cortical areas. In addition, the quality of signals recorded from any of the channels was determined by the computation of SNR, and we only included channels with SNR ≥ 3. This resulted in data included from 481 channels for V1 (224 in monkey 1, 257 in monkey 2) and 531 channels for V4 (306 in monkey 1, 225 in monkey 2).

Granger Causality analysis

To reduce computational time, the signals were down sampled to 128 time points at a sampling frequency of 254.34 Hz.

In its original formulation (*5*), GC between two times series $Y(t)$ and $X(t)$ is computed by fitting a multivariate vector autoregressive model (MVAR) with finite memory p. The fit consists in estimating the linear interaction coefficients $\mathbf{A}_{k,k=1\ldots p}$ by least squares regression, yielding residual fit error of mean zero and covariance $\mathbf{\Sigma}$.

Spectral GC is characterized at each frequency $\lambda \in [0, F_s/2]$ via the cross-spectral density matrix $\mathbf{S}(\lambda)$ and the MVAR transfer function matrix $\mathbf{H}(\lambda)$ yielding factorization $\mathbf{S}(\lambda) = \mathbf{H}(\lambda) \cdot \mathbf{\Sigma} \cdot \mathbf{H}(\lambda)^*$ (*6*). Spectral GC is then defined as:

$$f_{Y\to X}(\lambda) = \ln\left(\frac{|S_{xx}(\lambda)|}{\left|S_{xx}(\lambda) - H_{xy}(\lambda) \cdot \left(\Sigma_{yy} - \Sigma_{xy} \cdot \Sigma_{xx}^{-1} \cdot \Sigma_{xy}^{*}\right) \cdot H_{xy}^{*}(\lambda)\right|}\right), \ \lambda \in [0, F_S/2].$$

To provide a more refined measure of the communication between time series of LFPs in channels Y and X, in our analysis we computed the GC between Y and X conditioned on $\mathbf{Z}$ (called here Conditional GC, with acronym cGC).

This more refined measure discounts the possible confounding effect of time-lagged interactions mediated by activity of other recorded channels $\mathbf{Z} = [Z_1, \dots, Z_m]$ rather than direct communication between the two considered nodes Y and X (see below for details of our infomax partial conditioning choice of the m channels $\mathbf{Z}$).

Following (*7*), cGC $f_{Y\to X|\mathbf{Z}}$ was computed by first applying a reduced least-square autoregression to the time series $X, \mathbf{Z}$ only, yielding residual error time series $X^{\dagger}$, $\mathbf{Z}^{\dagger}$. Then, cGC was defined via the identity: $f_{Y\to X|\mathbf{Z}}(\lambda) = f_{(Y\oplus\mathbf{Z}^{\dagger})\to X^{\dagger}}(\lambda)$ $, \lambda \in [0, F_s/2]$, allowing to express cGC with the original definition as unconditional GC between the variables $(Y \oplus \mathbf{Z}^{\dagger}) = \binom{Y}{\mathbf{Z}^{\dagger}}$ and $X^{\dagger}$.

In our analysis, cGC was computed by the 'MVGC' method based on the computation of MVAR autocovariance sequences via Yule-Walker equations using the 'MVGC toolbox' (*8*). The magnitude of spectral GCs did not qualitatively vary using alternative methods such as matrix partitioning (*9*), nonparametric spectral factorization (*10*), or time reversed GC (*11*).

The stationarity of LFPs, an important check for the application of Granger analyses, was assessed by ensuring that the MVAR characteristic polynomial $\varphi_{\mathbf{A}_k}(z) = \left|\mathbf{I} - \sum_k \mathbf{A}_k z^k\right|$ was invertible within unit disc, i.e. that $\max\left\{1/|z|, z \in \mathbb{C} : \varphi_{\mathbf{A}_k}(z) = 0\right\}$ was always < 1 (*8*).

An important aspect of the analysis is the choice of which and how many (m parameter) the channels $\mathbf{Z}$ are chosen for conditioning in cGC. Conditioning on all available channels complementary to Y and X (m = ALL) ('full conditioning') might suffer from lack of sufficient data to estimate all autoregressive models needed for this calculation. In addition, full conditioning would make cGC unevenly scaled (since the number of available channels could vary across sessions), and regressing out channels $\mathbf{Z}$ within the same laminar compartments of Y or X would likely discount genuine interactions, because of the stronger correlations between channels within the same compartment due to intrinsic physical effects such as volume conduction. Following (*12*), we thus applied the infomax partial conditioning strategy. We considered for conditioning only channels outside the laminar compartments of channels X and Y.

We then chose $\mathbf{Z}$ to be the m channels with highest mutual information with Y and X (cGCs for different m are shown in Figure S7). Mutual information (*13*) between channel pairs was computed

on the Hilbert envelope of LFP time series demodulated in 2 Hz frequency bins, then integrated in time and frequency. We used the 'Information toolbox' (*14*) for mutual information estimation, and the method by (*15*) for subtracting the limited sampling bias.

We chose the free parameters of the conditioning (the number and identity of conditioning channels) as the ones with the best Akaike Information Criterion (AIC) and the autoregression coefficient of determination R^2 (Figures S7A-D). The two indices were adjusted to the size of the data sample to AICc (*16*) and R_c^2 (*17*) in order to prevent from data overfitting, though the correction did not affect the results much. The optimization of AICc and R_c^2 led us to the selection of an MVAR model with memory p=10 (≈40 ms, we tested $p \in [1, 16]$, i.e. $\approx [4, 65\text{ms}]$; p=10 yields asymptotical best R_c^2), and conditioning variable $\mathbf{Z}$ made by $m = 2$ channels outside the compartments of X and Y, achieving average R_c^2=0.8, S.E.M= 0.0006 (p=10 and m=2 (out) in Figure S7).

To estimate the statistical significance of the empirical cGCs and to exclude any possible residual limited sampling biases, we recomputed cGCs after randomly shuffling the data across trials (we used 100 different shuffles for each directed channel pair). The significance of empirical cGC magnitudes was then assessed by setting a significance threshold equal to the 95th percentile of shuffled cGCs (*9*).

Microsaccades and their relationship to LFP activity

Neural activity in visual cortex is influenced by the small fixational eye movements termed microsaccades (*18, 19*). Attentional modulation of neural activity in areas V4 and IT has furthermore been found to depend on whether microsaccades were directed towards the attended stimulus, as indicated by a spatial cue (*20*). It is therefore likely that LFPs are also influenced by microsaccades. We investigated the relationship between microsaccades and LFPs in order to determine whether microsaccades could account for the modulation of LFP dynamics during attention.

Microsaccades were detected as described previously (*21*). Briefly, we detected microsaccades by using the algorithm developed by Engbert and Kliegl (*22*). We converted eye position to velocity and low-pass filtered the velocity traces (*20*) at 20 Hz using a 2nd order Butterworth filter. An eye movement was classified as a microsaccade if the velocity is larger than a threshold for at least three consecutive time points. The threshold is set to six times the median estimator, given by: $\sqrt{median(x^2) - median(x)^2}$, where x is the eye position channel. Thus, the threshold is determined for each single trial. The use of the median estimator ensured that microsaccade detection is relatively robust to different levels of noise.

We detected microsaccades with an average amplitude of 0.47 ± 0.26° (mean ± standard deviation) and velocity of 17.72 ± 7.22 (Figure S13A). In line with previous studies (*21*), 96.1 % of detected microsaccades were smaller than 1° and they occurred on approximately 47.9 ± 3 % (mean ± SE) of trials (figure) with a rate of approximately 0.63 ± 0.04 Hz (Figure S13B-C). We found small

differences in the proportion of trials on which we detected microsaccades (repeated measures ANOVA; $F(2)=28.69$, $p < 0.001$) and the microsaccade rate (repeated measures ANOVA; $F(2)=21.38$, $p < 0.001$). Both these effects were driven by one of the attend away conditions (attend away 1), as this condition revealed both a higher proportion of trials with microsaccades ($p<0.001$, two-sided Wilcoxon signed rank tests) and a higher microsaccade rate ($p<0.01$, two-sided Wilcoxon signed rank tests) than the attend RF and attend away 2 condition. There was no difference in the trial proportion or rate between the attend RF and attend away 2 condition.

We next investigated the direction of microsaccades between attention conditions. We compared the microsaccade frequency between attend RF and attend away conditions across microsaccade directions (aligned to the RF location, corresponding to 0° in Figure S13D). Although the relative frequency of microsaccades between attend RF and away conditions differed across directions for each subject (chi-squared test, $p<0.001$), the pattern of this effect was markedly different across subjects. While microsaccades were more frequently directed away from the RF in monkey 1, they were more frequently directed towards the RF location for monkey 2 (chi-squared residuals test at 0.05 significance level with Bonferroni correction).

We tested whether the effect of microsaccades on LFP dynamics differed across attentional conditions. We investigated the relationship between microsaccades and LFP dynamics by computing the average LFP around the time of microsaccades for each of the attention conditions (Figure S13E-F). The LFP exhibited a negative deflection between approximately 30 ms and 200 ms after a microsaccade. However, this deflection did not differ across attentional conditions, as tested by a sliding window (20 ms window size) single factor (attention condition) repeated measures ANOVA across channels (corrected for multiple comparisons using FDR), indicating that the effect of microsaccades on the LFP dynamics was not modulated by attention. Finally, we verified that the spectral decomposition of the LFP revealed the same activity pattern on trials with and without microsaccades. Thus any differences in the LFP across attention conditions were independent of microsaccades.

Supplementary Materials

Data acquisition hardware

- Electrode probe (16 recording contacts, inter-contact spacing of 150 µm, model E16R-150-S1-L10, brand ATLAS Neuroengineering, Belgium);
- Hydraulic manipulator (model MO-97A, brand NASHIRIGE, Japan);
- Preamplifier (model HS36, Neuralynx, USA);
- CRT Monitor (size 22“, resolution 1280x1024 pixels, frame rate 120Hz, model HM204DTA, brand Iiyama, Japan);
- ViewPoint Eye tracker (model BHU03, brand Arrington research, USA);

- Digital acquisition tool (Digital Lynx: 32 channels, sampling frequency of 32556 Hz (~32 kHz), digital quantization resolution of 24 bits)

Data acquisition software

- Stimuli presentation and behavioral control: Remote Cortex 5.95
  (Laboratory of Neuropsychology, National Institute for Mental Health, Bethesda, MD).

- Raw signals extraction/filtering: Cheetah 5.6.3 (Neuralynx Inc., USA).

Data Analysis software

- MATLAB® 2018b (Mathworks Inc, USA);
- Neuralynx MATLAB-Netcom Utilities 6.0.0 (Neuralynx Inc., USA);
- Chronux Toolbox 2.12 chronux.org (*23*)
- MVGC Toolbox (*8*)
- Mutual Information toolbox (*14*)

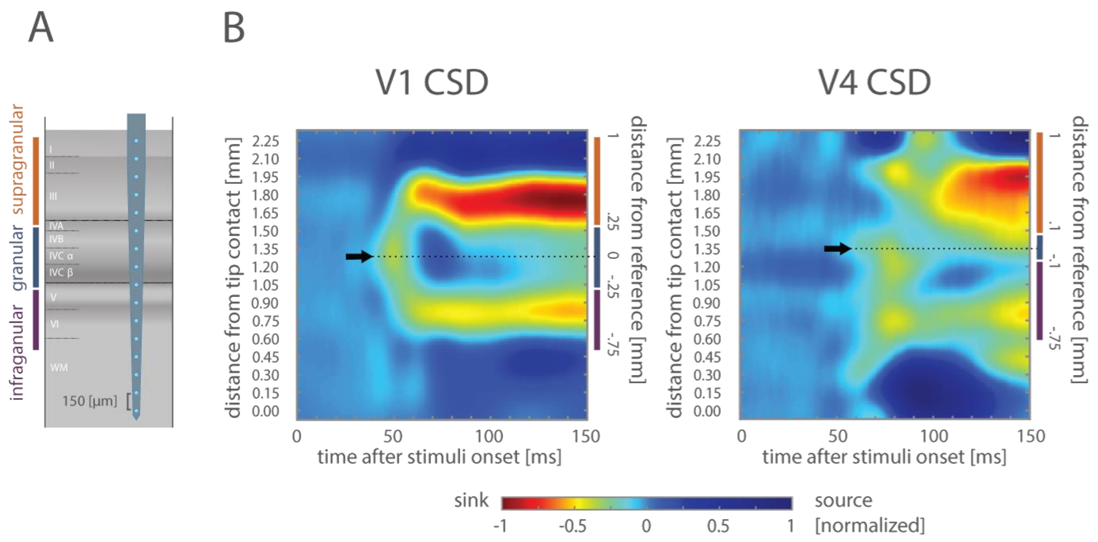

Fig. S1. Recording tools and Current Source Densities. A) Sketch to indicate targeted laminar recording sites. Probes (16 contacts, 150 µm contact spacing) were injected normal to the cortical surface, aiming to cover all layers. B) Stimulus-induced CSD (example session from monkey 1), for both V1 (left) and V4 (right). Earliest current sinks were identified as layer IV (black arrows). Based on their distance from reference depth, recording contacts were assigned to granular, infragranular and supragranular compartments (shown on the right of CSDs).

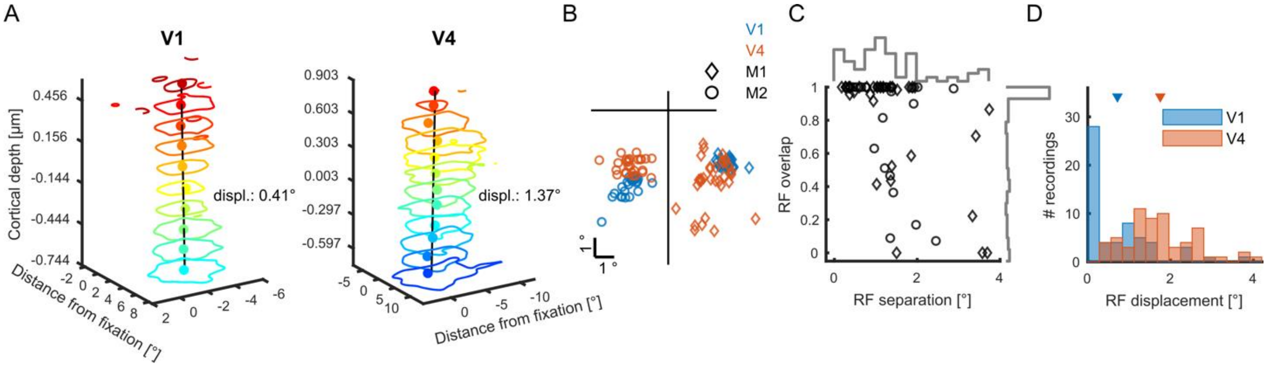

Fig. S2: Receptive field locations in the two monkeys. Receptive fields (RF) were obtained from MUAe activity for each electrode contact. RFs were mapped by a reverse correlation method (Methods) using a 0.5 or 1˚ sized stimulus for the V1 RFs and a 1 or 2˚ sized stimulus for the V4 receptive field. **A)** Example RFs. RF contours are drawn from z-scored response maps where z-score values exceeded 3. Color code indicates progression from more superficial to deeper contacts (red through yellow to blue). The black line depicts the fit (least-squares) to the x and y position across electrode contacts. The RF displacement (displ.) is computed as the geometric distance between the fitted RF location on the deepest and the most superficial channel. **B)** Average RF center locations (across channels) for each recording, separately for each subject (M1-M2) and area. **C)** RF separation between V1 and V4 plotted against their overlap, expressed as the proportion of the V1 RF. The histograms along the top (right) indicate the distribution of RF separation (overlap) across all recordings. **D)** RF displacement for V1 and V4 across all recordings. Note that the MUAe analysis and the relatively coarse spatial location sampling (0.5˚ or larger sized stimuli) results in RFs that are somewhat larger than those reported for V1 single units (*24*), but the main purpose for the current study was to get an estimate of RF centres and whether RF overlap existed between V1 and V4 locations, for which the employed method was sufficient.

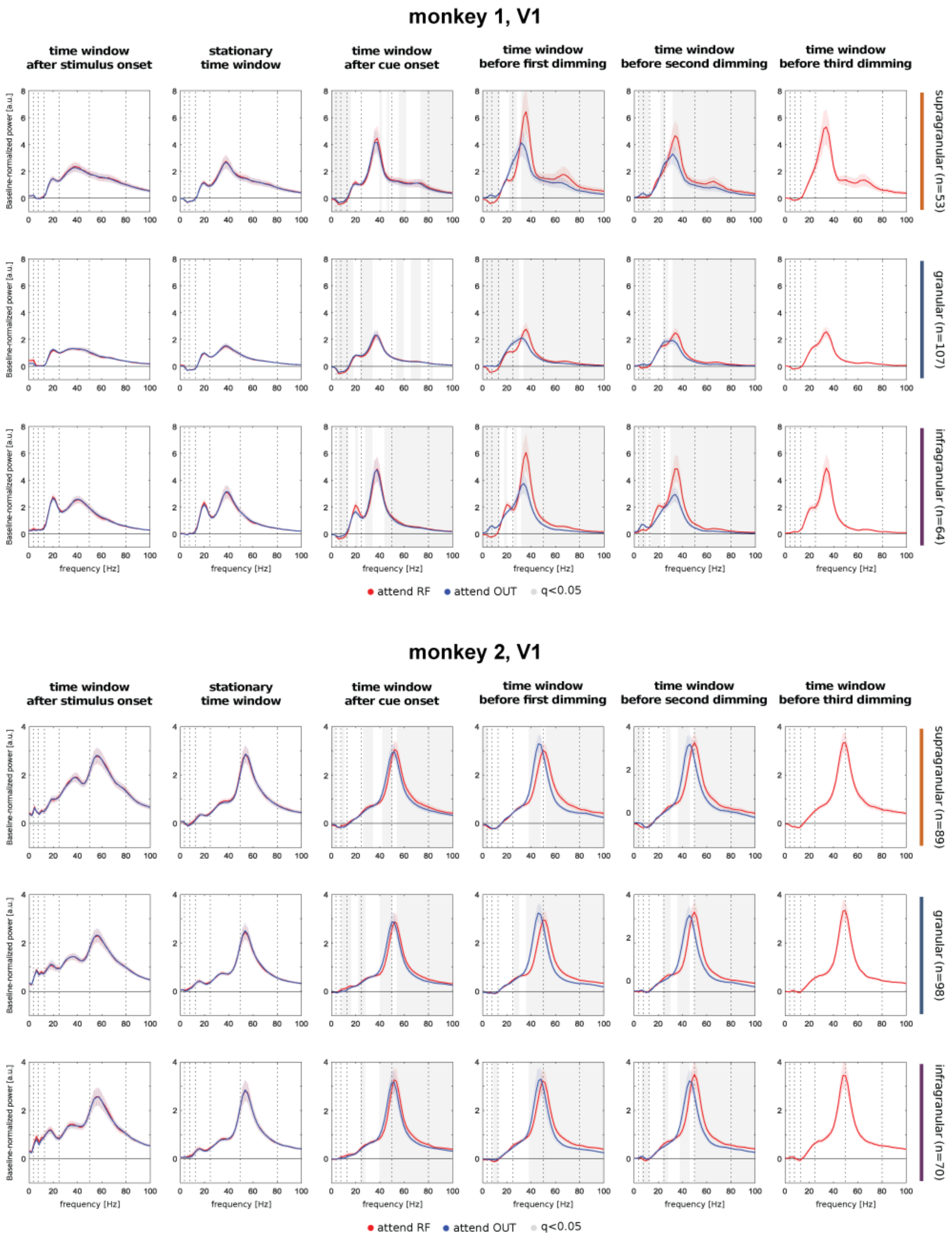

Fig. S3: Spectral power of V1 LFPs during the attentional task execution. Panels in each column show baseline-normalized spectral power (mean ± S.E.M across depths, sessions) computed in 503.25 ms (512 time points) task-related time windows (post-stimulus time window: 0 to 503.25 ms after stimulus onset; stationary time window: 200 to 703.25 ms after stimulus onset; 0 to 503.25 ms after cue onset; time windows before dimmings: -503.25 to 0 ms respectively before first, second and third dimming), in the two attentional conditions (attend RF, attend OUT). Baseline time window is set to -203.5 to 0 ms before stimulus onset. Panels with spectral power before

second and third dimming just include trials with unchanged contrast in RF, implying that stimulus at RF location did not dim at previous times. Panels in each row refer to different laminar compartments (supragranular, granular,

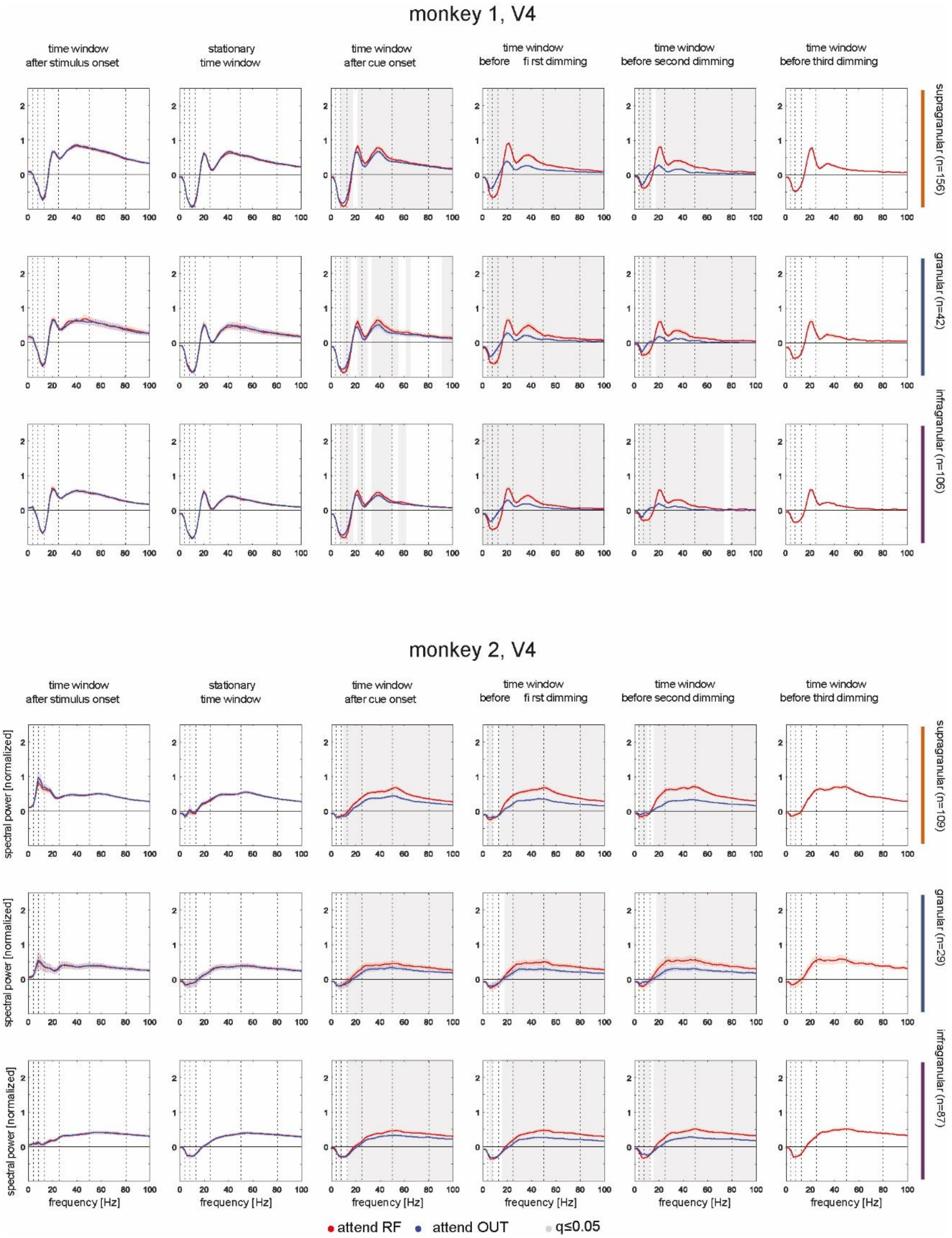

Fig. S4: Spectral power of V4 LFPs during the attentional task execution. Panels in each column show baseline-normalized spectral power (mean ± S.E.M across depths, sessions) computed in 503.25 ms (512 time points) task-related time windows (post-stimulus time window: 0 to 503.25 ms after stimulus onset; stationary time window: 200 to 703.25 ms after stimulus onset;

0 to 503.25 ms after cue onset; time windows before dimmings: -503.25 to 0 ms respectively before first, second and third dimming), in the two attentional conditions (attend RF, attend OUT). Baseline time window is set to -203.5 to 0 ms before stimulus onset. Panels with spectral power before second and third dimming just include trials with unchanged contrast in RF, implying that stimulus at RF location did not dim at previous times. Panels in each row refer to different laminar compartments (supragranular, granular, infragranular), for monkey 1 (three top rows) and monkey 2 (three bottom rows). Dashed lines report the spectral bands selected (theta 4-8 Hz, alpha 8-13 Hz, beta 13-25 Hz, low gamma 25-50 Hz, high gamma 50-80 Hz), gray shaded background is for significant differences between attentional conditions (two-sided Wilcoxon signed rank tests, FDR corrected, $q \leq 0.05$).

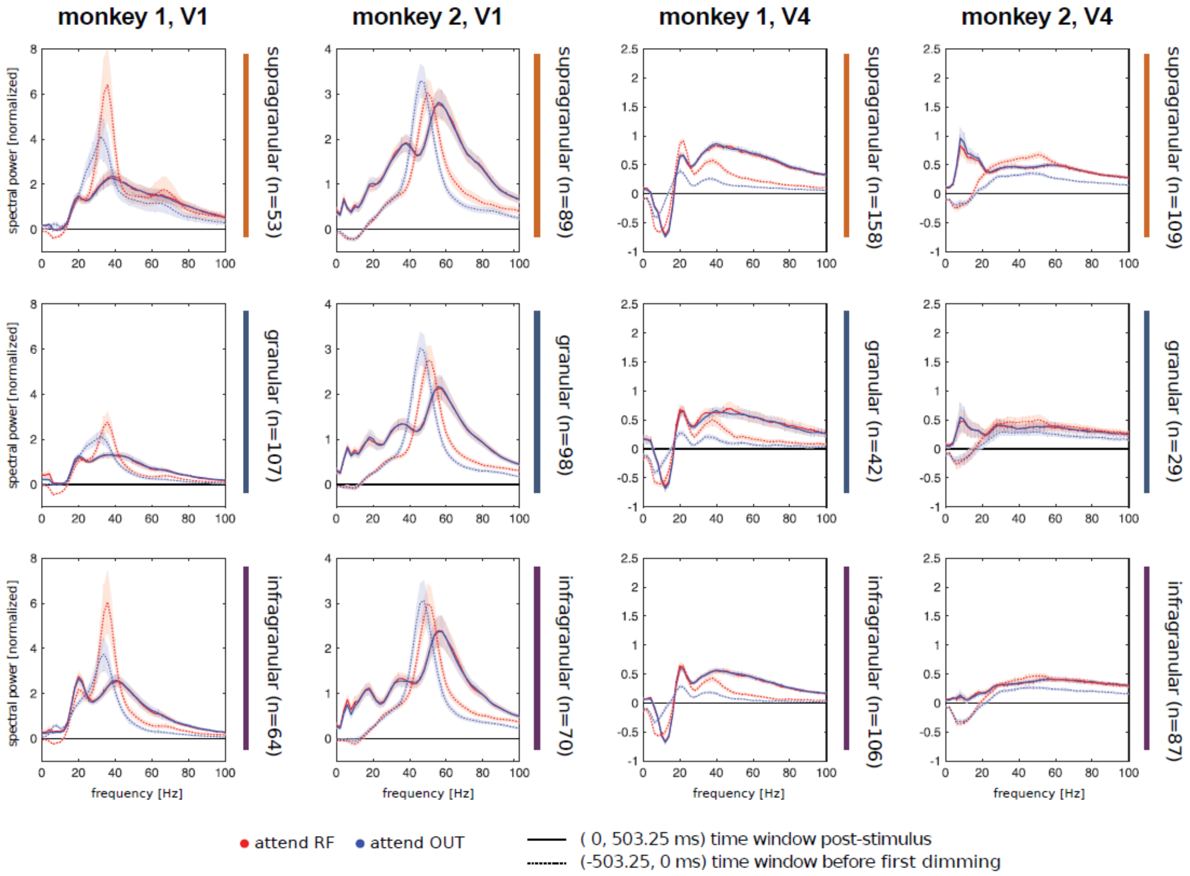

Fig. S5: Comparison of stimulus triggered LFP spectral power with attentional related spectral power. Panels in each column show baseline-corrected spectral power (mean ± S.E.M across sessions and depths) computed at different task-related time windows (solid lines show spectral power at times 0 to 503.25 ms after stimulus onset; dotted lines show spectral power at times -503.25 to 0 ms before first stimulus dimming), for the two attentional conditions (attend RF, attend OUT). Baseline time window is set to -203.5 to 0 ms before stimulus onset. Panels in each row refer to different laminar compartments (supragranular, granular, infragranular), for the two monkeys and for both V1 and V4.

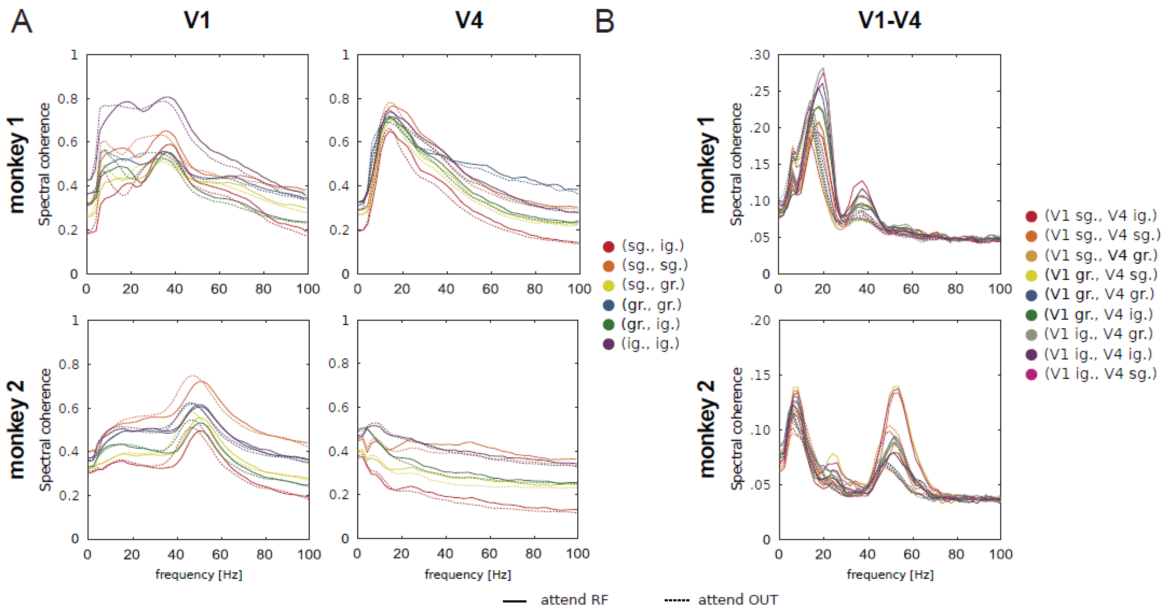

Fig. S6: LFP spectral coherence in the laminar compartments of V1 and V4 areas for the two monkeys. A) LFP spectral coherence (mean across sessions and depths) for monkey 1 (top row) and monkey 2 (bottom row), for paired laminar depths within visual area V1 (left column) and within V4 (right column). Spectral coherence is computed on LFPs at times -503.25 to 0 ms before first dimming and shown separately for trials with attention cued to RF location (solid lines) and for trials with attention cued OUT (dotted lines). **B)** same as in A, but for laminar depth pairs between V1 and V4 columns. **A-B)** Laminar compartment labels are shortened: sg. (supragranular), gr. (granular) and ig. (infragranular).

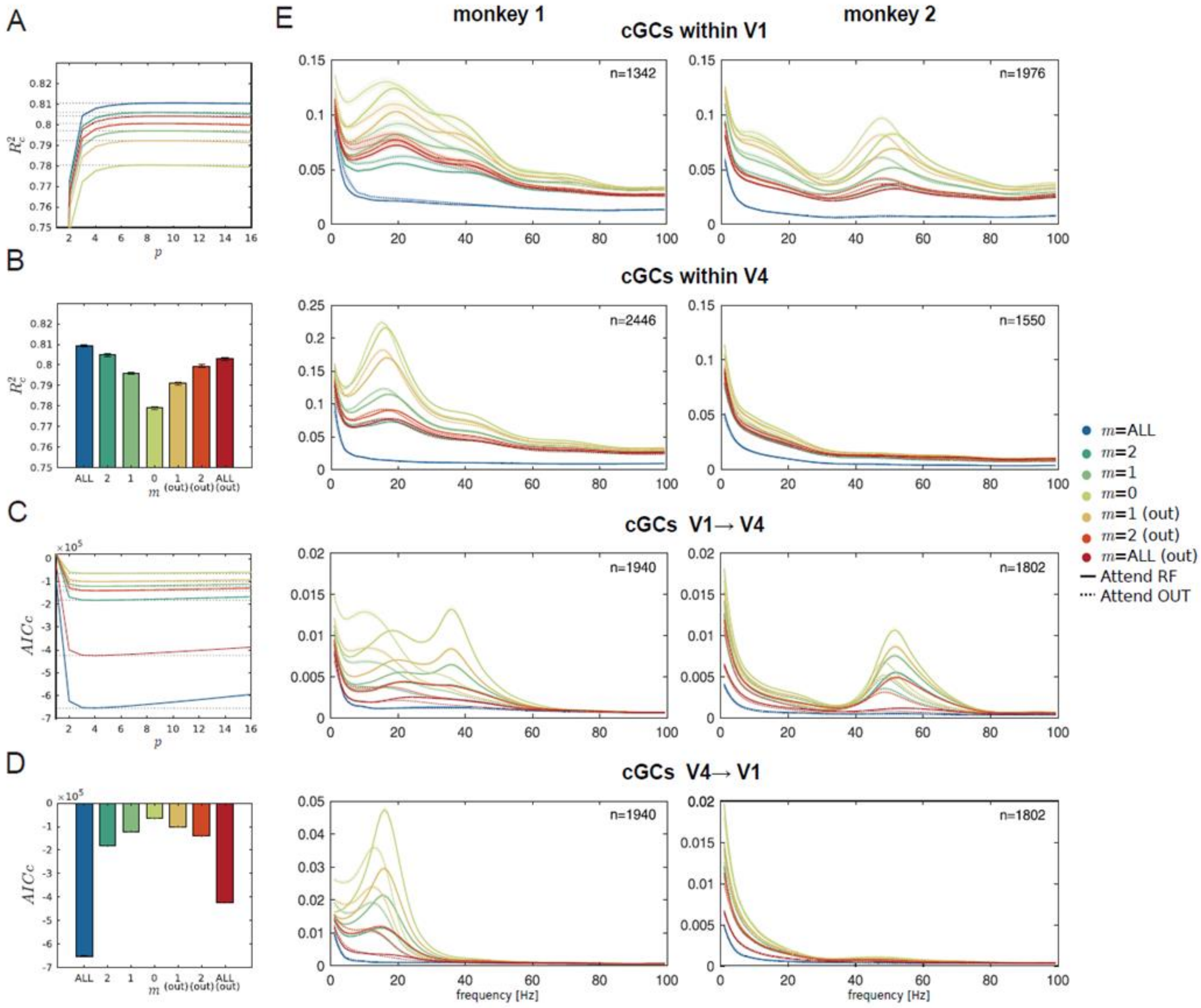

Fig. S7: Conditional GC estimation, selection of computational parameters p and m. A) Coefficient of determination Rc2 (mean ± S.E.M), a normalized measure of residual squared error in the estimation of VAR(p) models, adjusted to data set size. The VAR(p) models are estimated by least square QR decomposition for different values of VAR(p) model order p and for different conditioning strategies indexed by different values of the variable m. The values of Rc2 are pooled for all possible V1 and V4 depth pairs (within V1, within V4, and between V1 and V4) and for the two monkeys. B) Average Rc2 for different values of m and for fixed p=10, yielding overall best Rc2. C) Same as in A but showing Akaike Information Criterion corrected for data set size (AICc). D) Same as in B but showing AICc. E) Spectral cGCs (mean ± S.E.M) for all possible directed pairs within V1 (first row), within V4, (second row) and between V1 and V4 (from V1 to V4 in the third row, from V4 to V1 in the fourth row), for different values of m, and p=10. For the methods tested, cGCs did not drastically change when using different values of p, but spectral resolution could increase with larger p. Results are reported separately for monkey 1 (left panels) and monkey 2 (right panels), and for trials where attention was cued to RF location (solid lines) and OUT (dotted lines). A-E) Different conditioning strategies are indexed by different values of m: m=ALL (fully conditional cGC); m=1, m=2 (respectively computed by conditioning cGC to time series in 1 or 2 most informative contacts for any directed pair); m=1 (out), m=2 (out) and m=ALL (out) (respectively

computed by conditioning GC to time series in 1, 2 or ALL most informative contacts outside the laminar compartments of any directed pair).

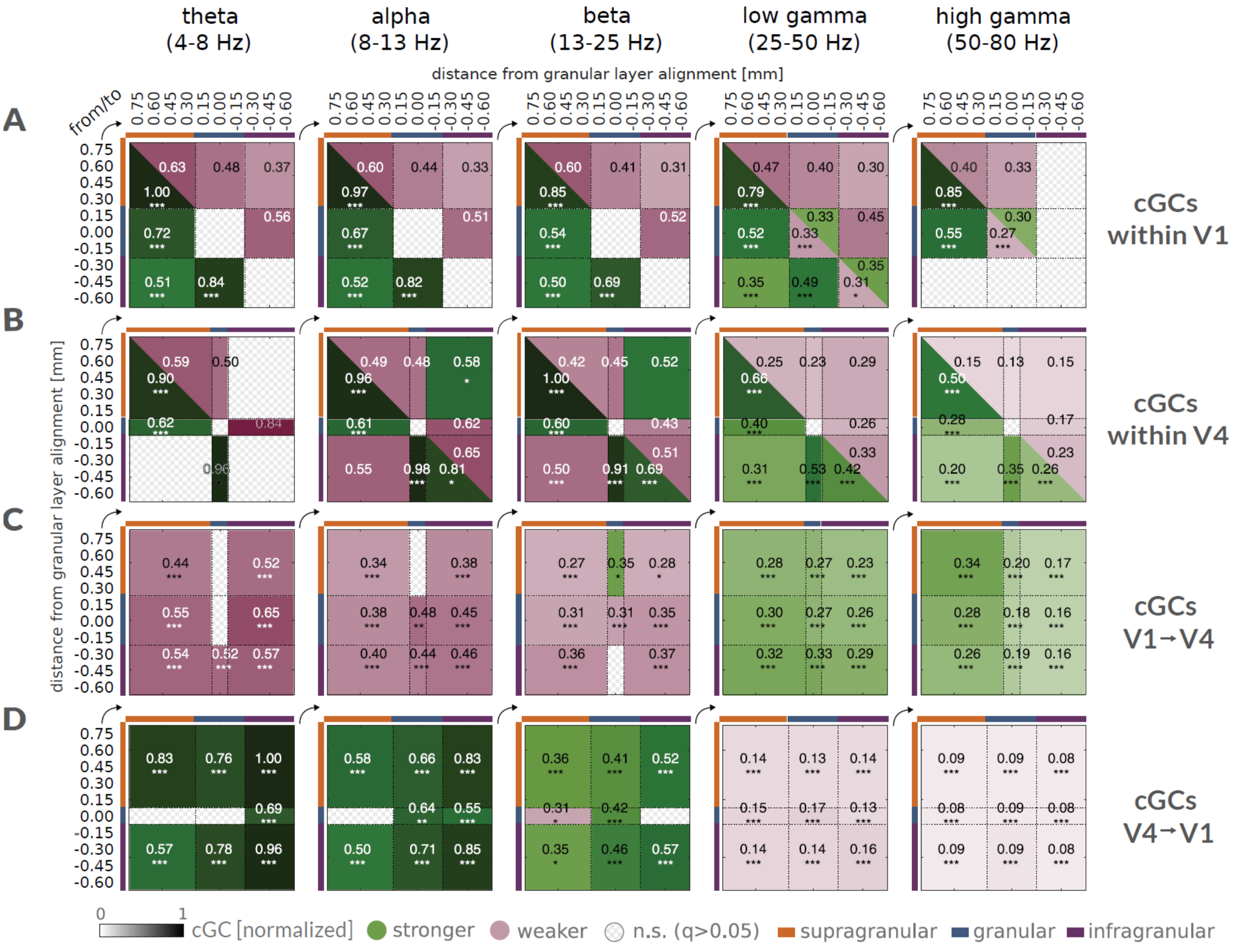

Fig. S8: Directed cGC connection matrices and dominant interactions. A) Directed connection matrices for cGCs (mean across sessions, mean among directed contact pairs in their respective laminar compartments, pooled for the two monkeys) within V1 columns, at different frequency bands (theta: 4-8Hz, alpha 8-13 Hz, beta 13-25 Hz, low-gamma 25-50 Hz, high-gamma 50-80 Hz). Connection matrices are color coded to show significantly dominant directions (green) and non-dominant directions (magenta). The color intensity of directed connections shows the relative strength of cGCs. Significance of cGCs dominance in opposite directions is assessed by two-sided Wilcoxon signed rank tests, FDR corrected within frequency bands (* indicates q ≤0.05, ** is for q ≤ 0.01, and *** is for q ≤0.001). **B)** Same as in A, but for cGCs within V4 columns. **C)** Same as in A, but for cGCs from V1 to V4 depths. **D)** Same as in A, but for cGCs from V4 to V1 depths.

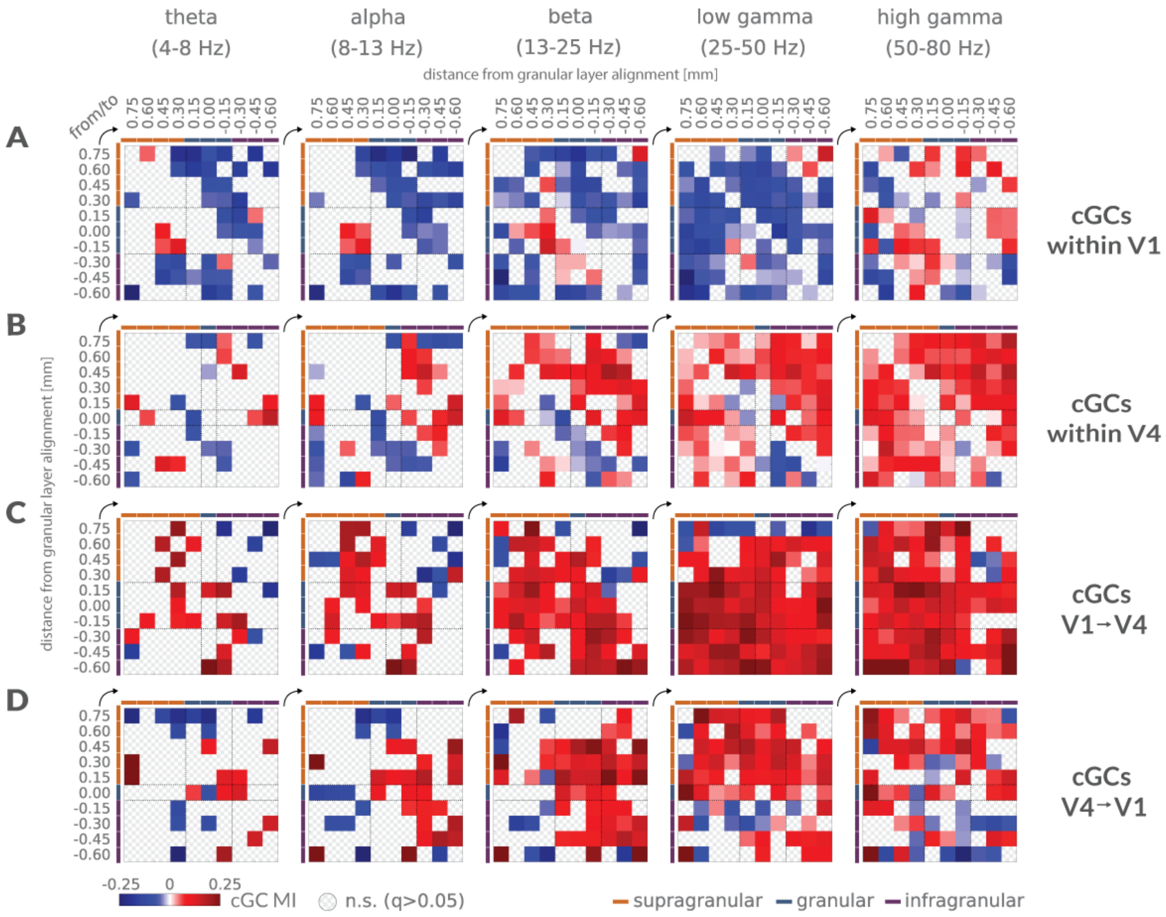

Fig. S9: Attentional modulation of directed cGCs. A) Directed connection matrices for significant attentional modulation index of cGC (cGC MI) among depth pairs within V1 columns (mean across sessions, pooled for the two monkeys), at different frequency bands (significance assessed via two-sided Wilcoxon signed rank tests, FDR corrected. q≤0.05 within frequency bands; theta: 4-8Hz, alpha: 8-13Hz, beta: 13-25Hz, low-gamma: 25-50 Hz, high-gamma: 50-80 Hz). B) Same as in A, but for V4 columns. C) Same as in A, but for cGCs MIs from V1 to V4 depths. D) Same as in A, but for cGCs MIs from V4 to V1 depths.

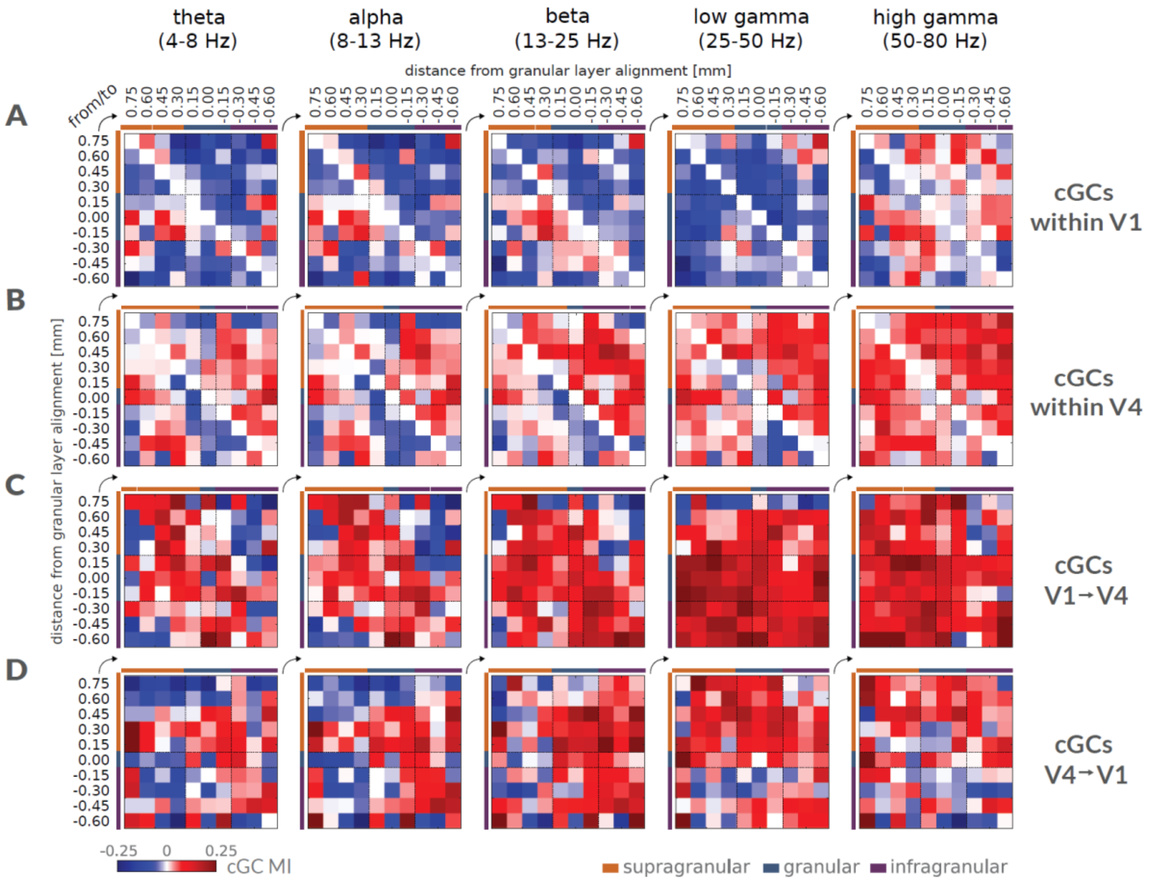

Fig. S10: Directed cGC connection matrices and attentional modulation (all modulations including non-significant). A) Directed connection matrices for cGC attentional MIs (mean across sessions, pooled for the two monkeys) among directed depth pairs within V1 columns, at different frequency bands (theta: 4-8Hz, alpha 8-13 Hz, beta 13-25 Hz, low-gamma 25-50 Hz, high-gamma 50-80 Hz). **B)** Same as in A, but for cGCs within V4 columns. **C)** Same as in A, but for cGCs from V1 to V4 depths. **D)** Same as in A, but for cGCs from V4 to V1 depths.

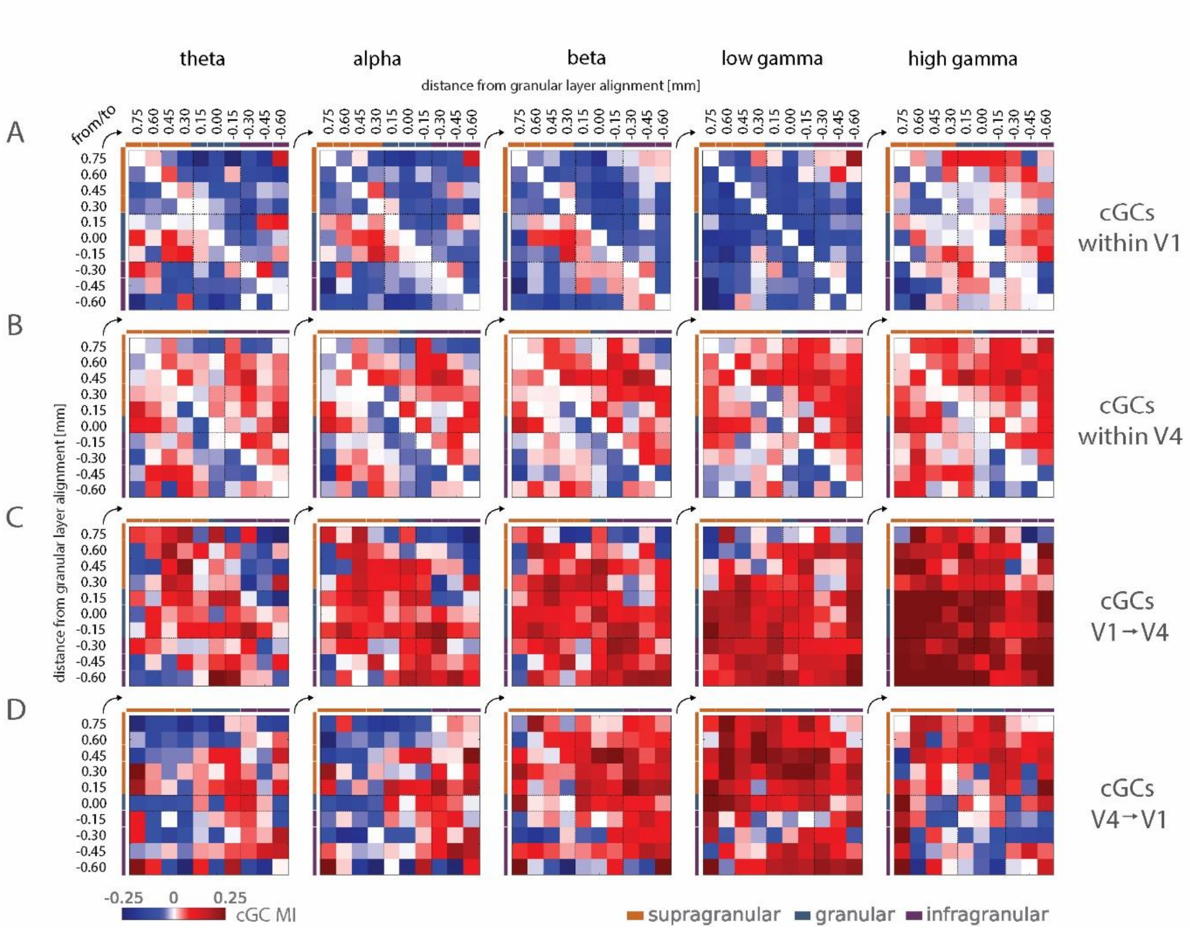

Fig. S11: Directed cGC connection matrices and attentional modulation for both monkeys with frequency bands adjusted to individualized spectral peaks. Bands for monkey 1 were defined as theta: 4-8Hz, alpha 8-13 Hz, beta 13-25 Hz, low-gamma 25-50 Hz, high-gamma 50-80 Hz; while for monkey 2 they were defined as theta: 7-12 Hz, alpha 18-22 Hz, beta 22-30 Hz, low-gamma 35-50 Hz, high-gamma 50-65 Hz. **A)** Directed connection matrices for cGC attentional MIs (mean across sessions, monkey 2) among directed depth pairs within V1 columns, at different frequency bands. **B)** Same as in A, but for cGCs within V4 columns. **C)** Same as in A, but for cGCs from V1 to V4 depths. **D)** Same as in A, but for cGCs from V4 to V1 depths.

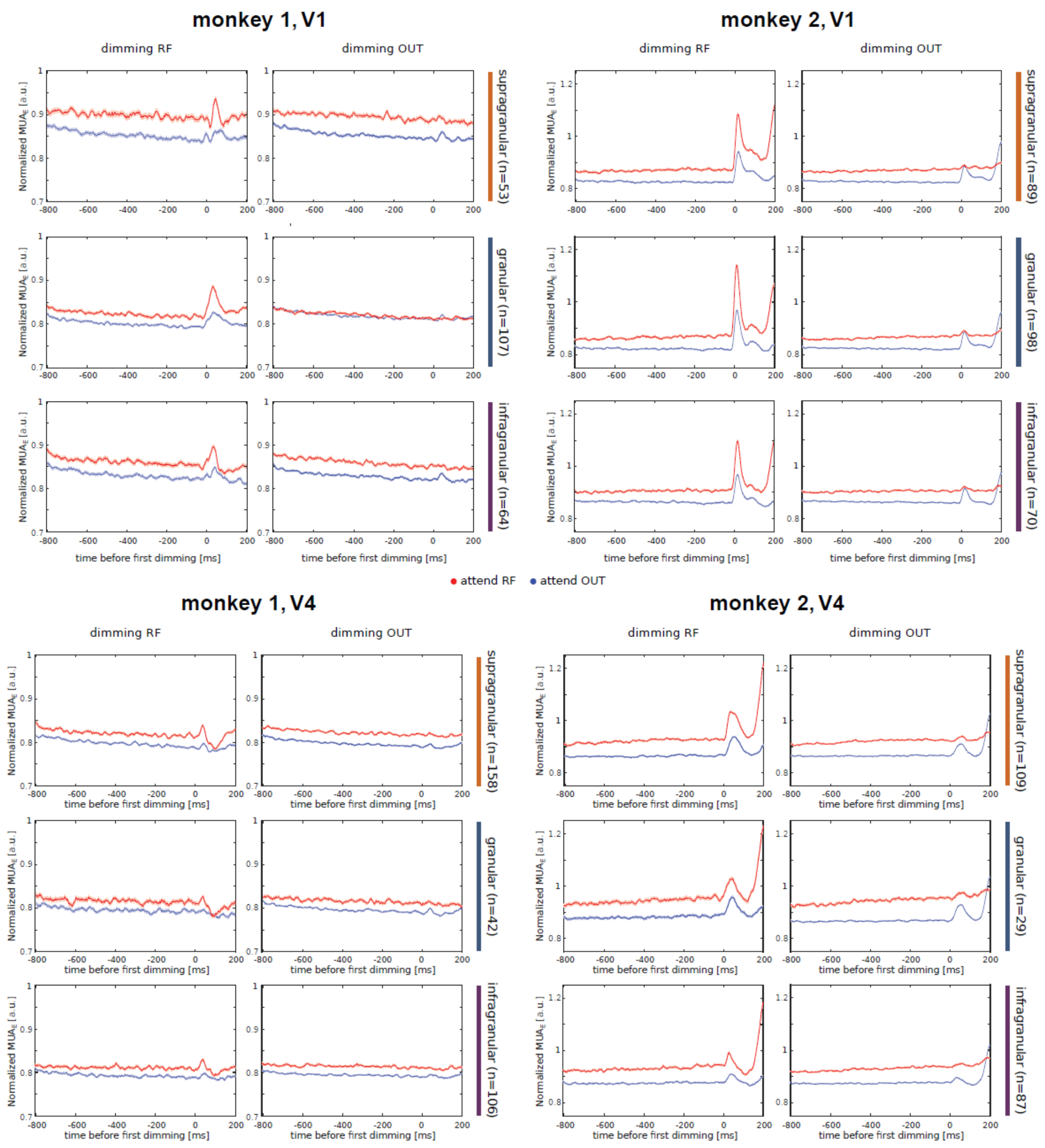

Fig. S12: Multi-unit activity during attentional task behaviour. Multi-unit activity envelope (MUAe; mean ± S.E.M across sessions and depths) aligned to the timing of first stimulus dimming, and normalized to (-200 to 0 ms) pre-stimulus activity. Results are shown separately for attentional conditions (attend RF, red lines and attend OUT, blue lines), dimming conditions (dimming RF, when first dimming occurred at RF location and dimming OUT, when first dimming occurred outside RF), and monkeys (monkey 1 two left columns, monkey 2 two right columns). Panels in different rows show results for different laminar compartments (supragranular, granular, infragranular) and for the two areas (V1 three top rows, V4 three bottom rows).

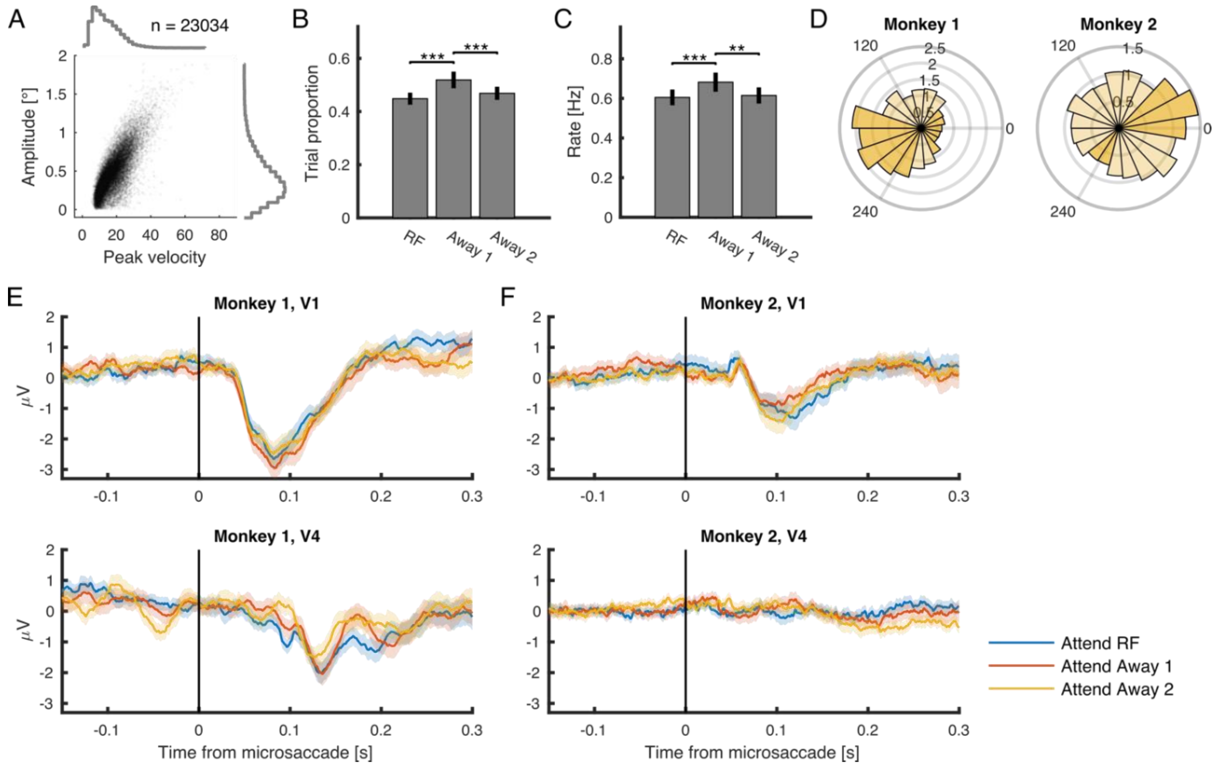

Fig. S13: Relationship between microsaccades and Local Field Potentials (LFP). A) Microsaccade amplitude versus velocity plot. The histograms along the top (right) indicate the distribution of velocities (amplitudes). **B)** Proportion of trials with microsaccades across attentional conditions. **C)** Microsaccade rate across attentional conditions. **D)** Relative microsaccade frequency between attend RF and attend away conditions for different microsaccade directions, aligned to the RF location (0°) for each subject. The relative frequency is computed for each direction (in 20° bins) as the ratio of the number of microsaccades between attention RF and OUT conditions. 0° corresponds to the RF location, which aligns with the attended stimulus in the attend RF condition. 120 and 240° respectively match the attend OUT1 and OUT2 stimulus locations. Directions with significantly higher relative microsaccade frequency are highlighted with darker color-fill (chi-squared residuals test at 0.05 significance level with Bonferroni correction). Microsaccades were not systematically directed towards or away from the RF location across subjects. **E-F)** Average LFP aligned to microsaccade onset. There was no statistical difference in the average LFP across attention conditions. Statistics: two-sided Wilcoxon signed rank test (panels B, C) and a repeated measures ANOVA (panels B, C, E and F). Data are represented as mean ± SEM across recordings (panel B and C) or channels (panel E and F); *, ** and *** respectively indicate significance levels of $p < 0.05$, $p < 0.01$ and $p < 0.001$.

Table S1.

Color specifications

Color	color code [R, G, B]			Luminance [cd/m^2]
**Gray (background)**	45	45	45	0.8
**Red**	220	0	0	12.8
**Green**	0	135	0	12.9
**Blue**	60	60	255	12.2
**Dimmed red**	140	0	0	4.2
**Dimmed green**	0	90	0	4.6
**Dimmed blue**	30	30	180	4.6

Color specifications and luminance for the stimuli used. Color code [RGB] indicates the red, green, blue lookup table locations for the respective stimuli.

Table S2.

Sessions, trials and behavioral performances.

	**monkey 1**			**monkey 2**			**monkeys 1&2**
	**V1**	**V4**	**V1&V4**	**V1**	**V4**	**V1&V4**	**V1&V4**
Sessions	34	35	34	30	30	28	62
Correct trials	15892	16301	15892	21632	21383	19852	35744
Error trials	806	818	806	443	384	362	1168
Total trials	16698	17119	16698	22075	21767	20214	36912
Performance (%)	95.17%	95.22%	95.17%	97.99%	98.24%	98.21%	96.84%
Missed (bar not released)	588	596	588	526	508	526	1114
Missed (fixation break)	1407	1460	1407	3397	3362	3397	4804

Number of sessions for data recorded in V1 and in V4. The columns V1&V4 report the number of sessions and trials where data were recorded simultaneously in both V1 and V4. Total number of task trials performed, number of task trials where attended stimulus was correctly reported, percentage of total trials resulting in correct behavior.

Table S3.

Selection of trials based on LFP signal energy.

	monkey 1			monkey 2			monkeys 1&2
	V1	V4	V1&V4	V1	V4	V1&V4	V1&V4
Number of selected trials	15594	16011	15468	21312	21120	19524	34992
Percentage Rejected	1.88%	1.78%	2.67%	1.48%	1.23%	1.65%	2.1%

Number of trials selected, and percentage of trials rejected in V1 and V4 by applying artefact removal criteria based on LFP signals energy. The columns V1&V4 report the number of simultaneous trials selected for both V1 and V4 once the trials selection was applied independently to V1 and V4.

Table S4.

Selection of channels based on SNR threshold.

		monkey 1		monkey 2	
		**V1**	**V4**	**V1**	**V4**
SNR ≥3	Total channels (chs/sessions)	224 (6.59)	306 (8.74)	257 (8.57)	225 (7.50)
	Supragranular	53 (1.56)	158 (4.51)	89 (2.97)	109 (3.63)
	Granular	107 (3.15)	42 (1.20)	98 (3.27)	29 (0.97)
	Infragranular	64 (1.88)	106 (3.03)	70 (2.33)	87 (2.90)

Total number of channels and average number of channels for each session in V1 and V4 selected by SNR ≥ 3 thresholding rule. Total number of channels selected, and average number of channels selected for each session in each V1 and V4 laminar compartment following laminar depth assignment.

SI References

1. A. Thiele, L. S. Delicato, M. J. Roberts, M. A. Gieselmann, A novel electrode-pipette design for simultaneous recording of extracellular spikes and iontophoretic drug application in awake behaving monkeys. *J Neurosci Methods* **158**, 207-211 (2006).
2. M. A. Gieselmann, A. Thiele, Comparison of spatial integration and surround suppression characteristics in spiking activity and the local field potential in macaque V1. *Eur J Neurosci* **28**, 447-459 (2008).
3. H. Super, P. R. Roelfsema, Chronic multiunit recordings in behaving animals: advantages and limitations. *Prog Brain Res* **147**, 263-282 (2005).
4. P. R. Roelfsema, M. Tolboom, P. S. Khayat, Different processing phases for features, figures, and selective attention in the primary visual cortex. *Neuron* **56**, 785-792 (2007).
5. C. W. J. Granger, Investigating causal relations by econometric models and cross-spectral methods. *Econometrica* **34**, 424–438 (1969).
6. J. F. Geweke, Measurement of Linear Dependence and Feedback Between Multiple Time Series. *Journal of the American Statistical Association* **77(378)**, 304–313 (1982).
7. J. F. Geweke, Measures of Conditional Linear Dependence and Feedback between Time Series. *Journal of the American Statistical Association* **79(388)**, 907–915 (1984).
8. L. Barnett, A. K. Seth, The MVGC multivariate Granger causality toolbox: a new approach to Granger-causal inference. *J Neurosci Methods* **223**, 50-68 (2014).
9. Y. Chen, S. L. Bressler, M. Ding, Frequency Decomposition of Conditional Granger Causality and Application to Multivariate Neural Field Potential Data. *Journal of Neuroscience Methods* **150(2)**, 228–237 (2006).
10. M. Dhamala, G. Rangarajan, M. Ding, Analyzing information flow in brain networks with nonparametric Granger causality. *Neuroimage* **41**, 354-362 (2008).
11. M. Vinck *et al.*, How to detect the Granger-causal flow direction in the presence of additive noise? *Neuroimage* **108**, 301-318 (2015).
12. D. Marinazzo, M. Pellicoro, S. Stramaglia, Causal information approach to partial conditioning in multivariate data sets. *Comput Math Methods Med* **2012**, 303601 (2012).
13. C. Shannon, A mathematical theory of communication. *Bell. Sys. Tech. J.* **27**, 379-423 (1948).
14. C. Magri, K. Whittingstall, V. Singh, N. K. Logothetis, S. Panzeri, A toolbox for the fast information analysis of multiple-site LFP, EEG and spike train recordings. *BMC Neurosci* **10**, 81 (2009).
15. S. Panzeri, A. Treves, Analytical estimates of limited sampling in different information measures. *Network* **7**, 87-107 (1996).
16. C. M. Hurvich, C. L. Tsai, Model selection for extended quasi-likelihood models in small samples. *Biometrics* **51**, 1077-1084 (1995).
17. H. Theil, *Economic Forecasts and Policy*. (North Holland Publishing Co. , 1961).
18. W. Bair, L. P. O'Keefe, The influence of fixational eye movements on the response of neurons in area MT of the macaque. *Vis Neurosci* **15**, 779-786. (1998).
19. D. A. Leopold, N. K. Logothetis, Microsaccades differentially modulate neural activity in the striate and extrastriate visual cortex. *Exp Brain Res* **123**, 341-345 (1998).
20. E. Lowet *et al.*, Enhanced Neural Processing by Covert Attention only during Microsaccades Directed toward the Attended Stimulus. *Neuron* **99**, 207-214 e203 (2018).
21. J. van Kempen *et al.*, Top-down coordination of local cortical state during selective attention. *Neuron*, (2021).
22. R. Engbert, R. Kliegl, Microsaccades uncover the orientation of covert attention. *Vision Res* **43**, 1035-1045 (2003).
23. H. Bokil, P. Andrews, J. E. Kulkarni, S. Mehta, P. P. Mitra, Chronux: a platform for analyzing neural signals. *J Neurosci Methods* **192**, 146-151 (2010).
24. M. Roberts, L. S. Delicato, J. Herrero, M. A. Gieselmann, A. Thiele, Attention alters spatial integration in macaque V1 in an eccentricity-dependent manner. *Nat Neurosci* **10**, 1483-1491 (2007).
